# Development of simplified probabilistic models for predicting phytoextraction timeframes of soil contaminants: demonstration at the DDX-contaminated Kolleberga tree nursery in Sweden

**DOI:** 10.1007/s11356-024-33858-x

**Published:** 2024-06-05

**Authors:** Paul Drenning, Anja Enell, Dan Berggren Kleja, Yevheniya Volchko, Jenny Norrman

**Affiliations:** 1 Department of Architecture and Civil Engineering, Chalmers University of Technology, 41296 Gothenburg, Sweden; 2 Swedish Geotechnical Institute (SGI), 58193 Linköping, Sweden; 3 Department of Soil and Environment, Swedish University of Agricultural Sciences (SLU), Box 7014, 75007 Uppsala, Sweden

**Keywords:** Phytoextraction, Dichlorodiphenyltrichloroethane (DDT), Probabilistic model, Uncertainty, Field experiment, Phytomanagement

## Abstract

Phytoextraction, utilizing plants to remove soil contaminants, is a promising approach for environmental remediation but its application is often limited due to the long time requirements. This study aims to develop simplified and user-friendly probabilistic models to estimate the time required for phytoextraction of contaminants while considering uncertainties. More specifically we: i) developed probabilistic models for time estimation, ii) applied these models using site-specific data from a field experiment testing pumpkin (*Cucurbita pepo* ssp*. pepo* cv. Howden) for phytoextraction of DDT and its metabolites (ΣDDX), iii) compared timeframes derived from site-specific data with literature-derived estimates, and iv) investigated model sensitivity and uncertainties through various modelling scenarios. The models indicate that phytoextraction with pumpkin to reduce the initial total concentration of ΣDDX in the soil (10 mg/kg _dw_) to acceptable levels (1 mg/kg _dw_) at the test site is infeasible within a reasonable timeframe, with time estimates ranging from 48–123 years based on literature data or 3 570–9 120 years with site-specific data using the linear or first-order exponential model, respectively. Our results suggest that phytoextraction may only be feasible at lower initial ΣDDX concentrations (< 5 mg/kg _dw_) for soil polishing and that alternative phytomanagement strategies should be considered for this test site to manage the bioavailable fraction of DDX in the soil. The simplified modes presented can be useful tools in the communication with site owners and stakeholders about time approximations for planning phytoextraction interventions, thereby improving the decision basis for phytomanagement of contaminated sites.

**Supplementary Information:**

The online version contains supplementary material available at 10.1007/s11356-024-33858-x.

## Introduction

### Background

Phytoextraction is the primary, and arguably the most well-known and thoroughly tested, phytotechnology for removing inorganic contaminants (and even for persistent organic pollutants such as PCBs and DDT (Denyes et al. 2013, 2016; Whitfield Åslund et al. 2008, 2010)) from soil by utilising the capacity of plants to take up bioavailable contaminants in the roots, transfer upwards and accumulate at higher concentrations in their harvestable tissues (Mench et al. 2010; Robinson et al. 2006; Vangronsveld et al. 2009). To do so, the plants function as 'bio-pumps' wherein they generate a flux (or plant-induced gradient) that drives water, solutes, and organic matter into the plant due to plant physiological demands and transpiration, which simultaneously enables active uptake of contaminants in the soil within the rooting zone (Herzig et al. 2014; Manzoni et al. 2011; Robinson et al. 2003a). There may not yet be a complete understanding of the physiological and biogeochemical mechanisms driving phytoextraction, but the process can be described in general terms as governed by two main variables: 1) contaminant concentration in harvestable plant tissue, usually indicated by the bioaccumulation factor, *BAF*, and 2) harvestable biomass production, *BMP* (Burges et al. 2018; Keller 2005; Keller et al. 2003; Robinson et al. 2015, 2009, 2006; Vangronsveld et al. 2009). The effectiveness of phytoextraction is, however, influenced by several, often site-specific, factors, such as the plant-available fraction of the contaminant in the soil, which is affected by soil biogeochemistry and the effects of spatial and temporal heterogeneity, and the growth of the plant, root density and distribution in the soil which may be impacted by the type of soil, physical and chemical conditions, climate factors, etc. (Burges et al. 2018; Keller 2005; Keller et al. 2003; Robinson et al. 2015, 2006; Van Nevel et al. 2007; Vangronsveld et al. 2009).

Several mathematical models to better understand the different mechanisms by which plants take up contaminants from soil and/or predict a timeframe for contaminant reduction via phytoextraction have been created by researchers from various backgrounds, which has led to several types of modelling approaches being tested, including: stochastic models that describe the mass balance of contaminant in the unsaturated zone subject to phytoremediation and leaching losses triggered by intense rainfall events varying over time (Manzoni et al. 2011); linear analytical equations that use empirical data to derive contaminant extraction potential from concentration in plant tissue and biomass production to estimate the decrease in soil concentrations over time (e.g., Algreen et al. 2014; Herzig et al. 2014; Robinson et al. 2009, 2006, 2003b; Van Nevel et al. 2007); dynamic systems models based on mechanistic plant physiology to take up (or volatilise) specific contaminants and predict phytoextraction timelines (e.g., Canales-Pastrana and Paredes 2013; Dehghani et al. 2021); different types of deterministic, mechanistic models that focus on comprehensively understanding specific phenomena in plant physiology, contaminant behaviour in soil and the mechanisms by which plant roots take up and transport contaminants into their biomass (e.g., Brennan and Shelley 1999; Davari et al. 2015; Gonnelli et al. 2000; Mathur 2004; Trapp and Matthies 1995; Verma et al. 2006; Vogeler et al. 2001); and more recently, machine learning analysis has been used to evaluate the factors that most influence metal uptake rates in phytoextraction (Shi et al. 2023).

Due to the many remaining limitations and uncertainties in applying phytoremediation, there is a need to clarify what time requirement is reasonable to expect in order to facilitate communication with stakeholders to promote their use in suitable situations. A main obstacle for phytoremediation generally is the perceived uncontrollability and difficulty to reliably estimate the timeframe of the remedial action for sufficiently reducing the risks, which is often focused on total concentrations in soils (Bleicher 2016). Many of the abovementioned phytoextraction models are mathematically intensive, mechanistic models that require specialized expertise and often entail many variables (some of which are difficult to obtain) that increases the complexity of the models (Canales-Pastrana and Paredes 2013; Terzaghi et al. 2018; Trapp and Matthies 1995), and may not be well-suited for estimating remediation timeframes.

Simplified models, with a low degree of complexity, such as analytical equations utilizing a linear steady-state extraction rate to describe the rate of phytoextraction, can be highly useful for preliminary calculations of time frames for phytoremediation. However, many models may not account for uncertainty and variability of the processes over time, which makes them less amenable for probabilistic risk analysis or feasibility studies (Manzoni et al. 2011). Accounting for the parameter uncertainties inherent in the analytical models by adopting a probabilistic approach may thus be a valuable modification to improve their robustness and provide clearer expectations regarding extraction potential, removal rates, and time requirements by presenting the most likely value (i.e., *mode*) and 90% uncertainty interval between the 5th and 95th percentile. To the best of our knowledge, this has not yet been done.

### Aim and objectives

The main aim of this study is to develop simplified and user-friendly probabilistic models to provide site owners and other stakeholders with a relevant approximation of the time requirement for phytoextraction of contaminants (e.g., metal(loid)s or POPs) to reach acceptable levels in soil, with due consideration to uncertainties. The specific objectives are to: i) develop simplified, probabilistic models for estimating the time required for phytoextraction; ii) test the models using data gathered from a field experiment to phytoextract ΣDDX with pumpkin (*Cucurbita pepo* ssp *pepo*); iii) contrast the time expectations using site-specific data to data derived from scientific literature; iv) investigate the sensitivity and uncertainties of the models by creating different modelling scenarios. Finally, the practical use of the suggested models is discussed including the implications for phytoextraction at the DDT contaminated Kolleberga site, a former tree nursery in Sweden.

## Materials and methods

### Field experiment

#### Site description

The field experiment site is the Kolleberga former tree nursery in the Scania region of Southern Sweden (Ljungbyhed) with fenced agricultural fields of ca. 23 hectares where pine and spruce trees were cultivated to serve the forestry industry. Since its initial usage in 1950s, technical DDT was used to control different types of pests, both by dipping the plants in barrels of dissolved DDT as well as spraying across the field by hand and with tractors. Despite a ban on DDT in the 1970s, DDT and its metabolites (including both *p,p’* and *o,p’* isomers) dichlorodiphenyldichloroethylene (DDE) and dichlorodiphenyldichloroethane (DDD), hereafter collectively referred to as ΣDDX, are still detected in the agricultural fields. The ΣDDX composition in the field soil is approximately 77% *p,p’*-DDT, 9% *o,p’*-DDT, 4% *p,p’*-DDD, 2% *o,p’*-DDD, 8% *p,p’*-DDE, and < 1% *o,p’*-DDE, which is similar to the makeup of technical DDT (ATSDR 2022) with marginally increased degradation products indicating that little degradation has occurred since its usage. Soil concentrations of ΣDDX, (*C*_*soil*_), at Kolleberga have been found to be in the range between 5–15 mg/kg _dw_ to a depth of approximately 0.35 m below ground level due to repeated ploughing and mixing of the soil in the fields (Nilsson 2019). These concentrations exceed the Swedish generic soil guideline value of acceptable levels for a less sensitive land use of 1 mg/kg _dw_ for the combined sum of *p,p’*- and *o,p’-* isomers of DDT, DDD, and DDE, i.e., ΣDDX.

The agricultural fields are no longer used for productive forestry but are managed by sowing a mixture of grasses, periodically cutting and ploughing the grass back into the soil. The site geology is loamy glacio-fluvial sand consisting of 87% fine-medium sand, 4% silt, 7% clay, and 2% gravel and larger stones, with a bulk density of approximately 1 500 kg/m^3^. The soil is well-drained and has moderate levels of organic carbon (1.6% TOC). Depth to the groundwater table is ca. 4–5 m.

#### Experimental set-up

As part of a 3-year field experiment, the effectiveness of pumpkin (*Cucurbita pepo* ssp. *pepo*) to remove ΣDDX from soil via phytoextraction was tested. The pumpkin family has been demonstrated to have the capacity to accumulate POPs, including ΣDDX and PCBs, at sufficiently high concentrations that phytoextraction of the contaminants may be a feasible remediation strategy (Denyes et al. 2016, 2013; Eevers et al. 2018; Kelsey and White 2005; Wang et al. 2004; White 2002, 2001). The specific cultivar selected for the field experiment, *C. pepo* cv. Howden, is a known accumulator of ΣDDX (Denyes et al. 2016; Lunney et al. 2010, 2004; Paul et al. 2015; White 2002, 2001; Whitfield Åslund et al. 2010).

The experiment was set up by first excavating soil with high content of ΣDDX (~ 10 mg/kg _dw_) from a selected part of the field to a depth of 35 cm and homogenizing. The experimental plots were established in triplicate in 2 × 2 m plots with a depth of 35 cm filled with the homogenized soil. In the first year, pumpkin seeds were sown directly into the plots using two seeds every 0.5 m in 4 rows in order to grow 4 plants per square meter, according to (White 2009). In the second year, to improve the pumpkin growth, the pumpkin seeds were first planted in pots in soil taken from the plots and allowed to develop into larger seedlings in a greenhouse for 4 weeks before being transferred to the plots. See the SM for more details.

#### Soil and plant sampling and analysis

The mean *C*_*soil*_ in the experimental plots at the start of the experiment was 10.5 ± 0.5 mg_DDX_/kg_soil_ _dw_ (see Table S1 in SM for concentrations of specific metabolites). After 16 weeks of growth, sampling points were randomized to select four sampling locations. Soil samples were collected using a core sampler (Φ 2cm) and extracting 20 soil cores around the randomly selected plants, to a depth of 20-25cm. The composite soil sample was homogenized and sieved (Φ 2mm) before analysis. Plants at the same locations were harvested by clipping the stem as close to the ground level as possible then separating the different plant parts (stems, leaves, and fruit) and weighing each individually. Roots were also collected at the same locations and weighed (after shaking and brushing loose the adhered soil). Representative samples of each plant part were collected in bags and sent for analysis of DDX concentrations in the different plant parts: *C*_*leaves*,_ *C*_*stems*_, and *C*_*roots*_*,* (g_DDX_/kg_biomass_ _dw_). Fruits were not included in this study due to insufficient literature data and low fruit production at Kolleberga.

Accredited commercial and university labs were contracted to perform soil and plant analysis for the field experiment. *C*_*soil*_ were measured using GC–MS according to (Rashid et al. 2010) and moisture content in soil and plant-parts by thermogravimetry according to EN 12880:2000. Prior to analysis, all plant parts were washed by rinsing and submerging in deionized water for 12 h. The washed samples were cut into small pieces, mixed to create a representative sample then placed in aluminium moulds and oven-dried at 35–37°C. The dried samples were ground into a fine powder of which 3g was weighed into a 50mL polypropylene tube and chemically extracted with n-Hexane and Diethyl ether. The extract was cleaned onto a silica gel/sulfuric acid column and ΣDDX quantified in the different plant parts using gas chromatography with electron capture detector (GC-ECD), modified from the Swedish Food Agency’s method for analysis of chlorinated pesticides and PCBs in food of animal origin, human milk and blood serum (SLV K3-25, version 4).

### Probabilistic phytoextraction models

Two probabilistic phytoextraction models for estimating time requirements for phytoextraction were developed in this study. They are based on existing analytical models that use simplified equations (Robinson et al. 2015, 2009, 2006, 2003b) combined with empirical data derived from literature and the field experiment at Kolleberga. To account for uncertainties and the inherent variability in phytoextraction, probability distributions were assigned to the two main aggregated input variables: the bioaccumulation factor (*BAF*) and the harvestable biomass production (*BMP*). The two tested models considered either a) a linear steady-state extraction over time, or b) a first-order exponential decay function.

#### Linear analytical model

The linear analytical models that are commonly used to provide an initial estimate of the time required for phytoextraction are based on a set of equations using empirical, easily acquired data. The standard equations vary somewhat between studies but in general are built on the assumption that the input variables are steady-state, i.e., plant uptake of contaminants and biomass production held constant over time. This results in a constant contaminant extraction potential (*E*), i.e., contaminant mass taken up per year, which can be used to calculate a mass balance for a certain amount of soil and estimate how many years are required to reduce the initial soil concentration to a final target level (Algreen et al. 2014; Grignet et al. 2020; Herzig et al. 2014; Robinson et al. 2015; Thijs et al. 2018). Robinson et al. (2015, 2009, 2006, 2003b) provided a series of equations using a limited number of variables for calculating the total metal uptake over time, which are used here as a starting point and slightly modified to assess DDX phytoextraction.

The contaminant extraction potential, *E* (mg_DDX_/year) is calculated based on the concentration of DDX in dry weight (dw) harvestable plant parts (i.e., stems and leaves), *C*_*plant*_ (mg_DDX_/kg_biomass_ _dw_) and the dry weight biomass production per harvest, year in this case, *BMP* (kg_biomass_ _dw_/year):

1 $$E = {C}_{plant}*BMP$$

The concentration in the different harvestable pumpkin parts is calculated using the initial concentration of DDX in soil, *C*_*soil,i*_ (mg_DDX_/kg_soil_ _dw_), and their respective bioaccumulation factors, *BAF* (mg_DDX_/kg_plant dw_ / mg_DDX_/kg_soil dw_):

2 $$BAF= \frac{{C}_{plant}}{{C}_{soil}}$$

Consequently, the extraction potential *E* is:

3 $$E= \left({BAF}_{stem}* {C}_{soil,i}\right)* {BMP}_{stem}+\left({BAF}_{leaves}*{C}_{soil,i}\right)* {BMP}_{leaves}$$

Assuming *E* to be constant over time, the corresponding remediation time, *t*_*final*_ (years) required to reduce the initial contaminant concentration in soil to a final target level is calculated as a constant (linear) decrease over time:

4 $${t}_{final}= \frac{{m}_{soil,i} - {m}_{soil,f}}{E}$$

where *m*_*soil,i*_ and *m*_*soil,f*_ are the total DDX mass in the soil (mg_DDX_) at the starting point of the phytoextraction (initial mass, *i*) and at the point when reaching the final target concentration (final mass, *f*). The mass of DDX, *m*_*soil*_, is calculated as:

5 $${m}_{soil}=\rho *V*{C}_{soil}$$

where *ρ* is the soil bulk density (kg_soil_/m^3^) and *V* is the volume of soil (m^3^) undergoing phytoextraction.

The removal rate, *k* (removal percentage/year) is then calculated as:

6 $$k= \frac{E}{{m}_{soil, i}}*100$$

The *BMP*, the treated soil volume (*V*), and the resulting mass of contaminants (*m*), and thus *E*, are in this case calculated for a unit area of 1 m^2^, a depth of 0.35 m and a soil bulk density of 1 500 kg/m^3^.

This linear analytical model with a constant extraction potential, *E*, and consequently a constant removal rate,* k*, does not account for variability or potential decreases in effectiveness and bioavailability over time (Robinson et al. 2015), and thus provides the theoretically shortest possible time for phytoextraction.

#### First-order exponential decay analytical model

Contaminant uptake is a function of the extractable contaminant mass in the soil, which would decrease over time thereby reducing effectiveness. This can be mathematically described using a first-order exponential decay function that could, at least theoretically, account for more complex soil chemistry and a decreasing pool of contaminants over time. Although the first-order decay model is commonly used to model biological degradation, it is important to note that the mechanism of reduction simulated here is only the removal by phytoextraction, not biological degradation. The analytical model becomes:

7 $${m}_{soil}(t)= {m}_{soil, i}*{e}^{-k*t}$$

where *m*_*soil*_ (*t*) is the contaminant mass (mg_DDX_) in soil at time *t* (years), *m*_*soil,i*_ is the initial contaminant mass (mg_DDX_) in soil, and *k* is the constant removal rate (%/year, Eq. 6). By rearranging Eq. 7, the corresponding remediation time, *t*_*final*_ (years), required to reduce the initial contaminant mass in soil to a final target mass, *m*_*soil,f*_ (mg_DDX_), is calculated accordingly:

8 $${t}_{final}=\frac{\mathit{ln}\left(\frac{{m}_{soil, i}}{{m}_{soil, f}}\right)}{k}$$

#### Probabilistic modelling

The two probabilistic phytoextraction models were set up in MS Excel using Eq. 2–8 and the Palisade add-in software @Risk 8.2 for defining probability distributions that represent uncertainties in the input variables (Table S2 and Figures S3-S10 in SM). Monte Carlo simulations were run 10 000 times by repeatedly picking random values from the probability distributions of input variables as described by Bedford and Cooke (2001), to calculate the probable extraction potential, removal rates and time requirements to reach set soil target values (Bedford and Cooke 2001).

### Input data and probability distributions

#### Site-specific data

Site-specific data, including ΣDDX concentrations and soil bulk density used in the models were derived from the field experiment in Kolleberga (Table 1). Empirical data from the first two years of phytoextraction using pumpkin (*Cucurbita pepo*) for phytoextraction of ΣDDX were used to calculate ranges and mean values for the two main variables: 1) *BAF* – calculated for each different harvestable plant tissue, and 2) *BMP* – in grams of dry weight per unit area. *BAF*s for the individual plant parts (roots, stems, leaves) were calculated using the mean soil ΣDDX concentration. Due to a Spanish slug infestation consuming the second year’s harvest, the raw data was adjusted to extrapolate the potential uptake of ΣDDX based on the root *BAF* and the translocation factor (*TF* = *C*_*stem*_*/C*_*root*_) derived from the first year’s data (more details in SM, Table S3).

**Table 1:** Input data: site-specific and derived from literature. Input data are mean values ± standard deviation; BAF [(mg/kg _plant dw_) / (mg/kg _soil dw_)], BMP [kg _biomass dw_ / (m^2^ year)]

Input data					Comment/Reference
	*BAF – ΣDDX*		*BMP*		
Dataset	*BAF*_*stem*_	*BAF*_*leaves*_	*BMP*_*stems*_	*BMP*_*leaves*_	
Site-specific (n = 6)	0.89 ± 0.46	0.13 ± 0.08	0.0783 ± 0.0416	0.190 ± 0.0808	Data from triplicate first 2 years of field experiment at Kolleberga
Literature (n = 15)	5.74 ± 3.35	0.435 ± 0.203	0.799 ± 0.690	0.410 ± 0.312	(Denyes et al. 2016; Lunney et al. 2010, 2004; Paul et al. 2015; White 2002, 2001; Whitfield Åslund et al. 2010)

#### Literature data

Empirical data from phytoextraction field experiments using pumpkin (*Cucurbita pepo* ssp. *pepo*, cv. Howden) to phytoextract aged ΣDDX (or specific metabolites) was gathered from a literature review in the Scopus database for field experiments using *C. pepo* to phytoextract DDX to create a representative dataset for both *BAF* and *BMP* (Table 1). Data for plant uptake derived from literature is often reported as total uptake (mg) or concentration (mg/kg _dw_) that is dependent on the specific field conditions and initial soil contaminant concentrations and not well-suited for providing generalised estimations. Instead, where applicable, the data were converted to *BAF*, which can be used as a more universal indicator of the potential contaminant accumulation capacity of a specific plant species in different types of contaminated soils. Similarly, two datasets for *BMP*_*stem*s_ and *BMP*_*leave*s_, were derived from literature and the estimated *BMP* per unit area (1 m^2^) calculated using the planting density in the field experiment, i.e., 4 plants per m^2^.

#### Modelling uncertainty

Probability distributions were created for each variable in both the experimental and literature dataset by assigning a Beta-PERT or Normal distribution to the input data in the @Risk software based on minimum, maximum and most likely values, or mean and standard deviation, respectively (see Figures S3-S10 in SM). By generating a range of probable values for the *BAF* and *BMP*, the probability distributions account for, at least partly, the inherent variability in plant uptake of contaminants and growth over time due to factors such as e.g., spatial and temporal heterogeneity, contaminant phytoavailability, complex soil biogeochemistry, soil, site and climatic conditions, root contact and absorption. Variability or decreases in effectiveness due to such factors that are difficult to predict can be approximated within the bounds of a probability distribution.

For investigating the sensitivity of the input variables in the probabilistic models, Spearman rank correlation coefficients were calculated by @Risk 8.2 (Palisade) to identify the variables that contributed most to the uncertainty in the model results. The sensitivity data are not extensively discussed under results but presented in SM.

### Output

#### Comparing models and datasets

Due to the different modelling approaches presented here, a main output is to compare the predicted time requirements as calculated using either the linear or first-order exponential decay model. Also, another point of comparison is the calculated extraction potential, removal rates and time requirements that differ based on either site-specific data or data derived from literature. Using site-specific data, the extraction effectiveness for specific DDT metabolites that together constitute more than 90% of the ΣDDX at Kolleberga, including *p,p’*-DDT, *o,p’-*DDT, *p,p’-*DDD, *p,p’-* DDE, is also analysed.

#### Scenario analysis

In addition, multiple model ‘scenarios’ were defined to further investigate the feasibility of phytoextraction and potential changes in removal rates and expected time requirements by testing different model assumptions in both the linear analytical model and first-order exponential decay model. The following scenarios were analysed (Table 2):

- different initial concentration of ΣDDX, *C*_*soil,i*_*,* and factoring in a phytoextraction ‘efficiency gradient’(= E * %_*eff*_*)*, which is an additional multiplication factor as a percentage of the simulated extraction potential. Varying with *C*_*soil,i*_, a gradient was used to model 100% efficiency for a ‘moderate’ concentration of 5 mg/kg _dw_ with increasing efficiency at ‘lower’ *C*_*soil,i*_ and decreasing efficiency at ‘higher’ *C*_*soil,i*_;
- simulating different values for *BAF*_*stem*_ to determine the minimum *BAF*_*stem*_ required to reduce *C*_*soil,i*_ to the target level within 25 years, which could be considered a reasonable time frame for phytoextraction; and
- optimised *BMP* to estimate changes in expected time requirements using site-specific *BAF* but literature-derived *BMP*.

**Table 2:** The different modelling scenarios considered in the study

*Scenario A*	*C*_*soil,i*_	Efficiency factor (*%*_*eff*_)	
Different initial concentrations, *C*_*soil,i*_: 10 – 2 (mg/kg _dw_), with a phytoextraction ‘efficiency gradient, 33 – 155% (*%*_*eff*_) to account for varying uptake in plants at different DDX concentrations	10	33%	Efficiency gradient based on data from (Paul et al. 2015) for ‘high’ (10.2 mg kg^−1^_dw_), ‘moderate’ (5.08 mg kg^−1^_dw_) and ‘low’ (0.291 mg kg^−1^_dw_) ΣDDX concentrations
	9	47%	
	8	60%	
	7	73%	
	6	87%	
	5	100%	
	4	118%	
	3	136%	
	2	155%	
*Scenario B*	*BAF*_*stem*_		
Simulations testing removal rates with different *BAF*_*stem*_ values	8, 10, 12, 14, 16, or 18		*BAF*_*stem*_ for ΣDDX extraction by pumpkin
*Scenario C*	*BMP*_*stem*_* & BMP*_*leaves*_		
Different amounts of BMP (kg_biomass,dw_ /year)	*BMP*_*exp*_ = *BMP*_*lit*_		Optimised *BMP*_*exp*_ based on literature data

## Results

### Linear versus first-order exponential decay phytoextraction

**Fig. 1:**
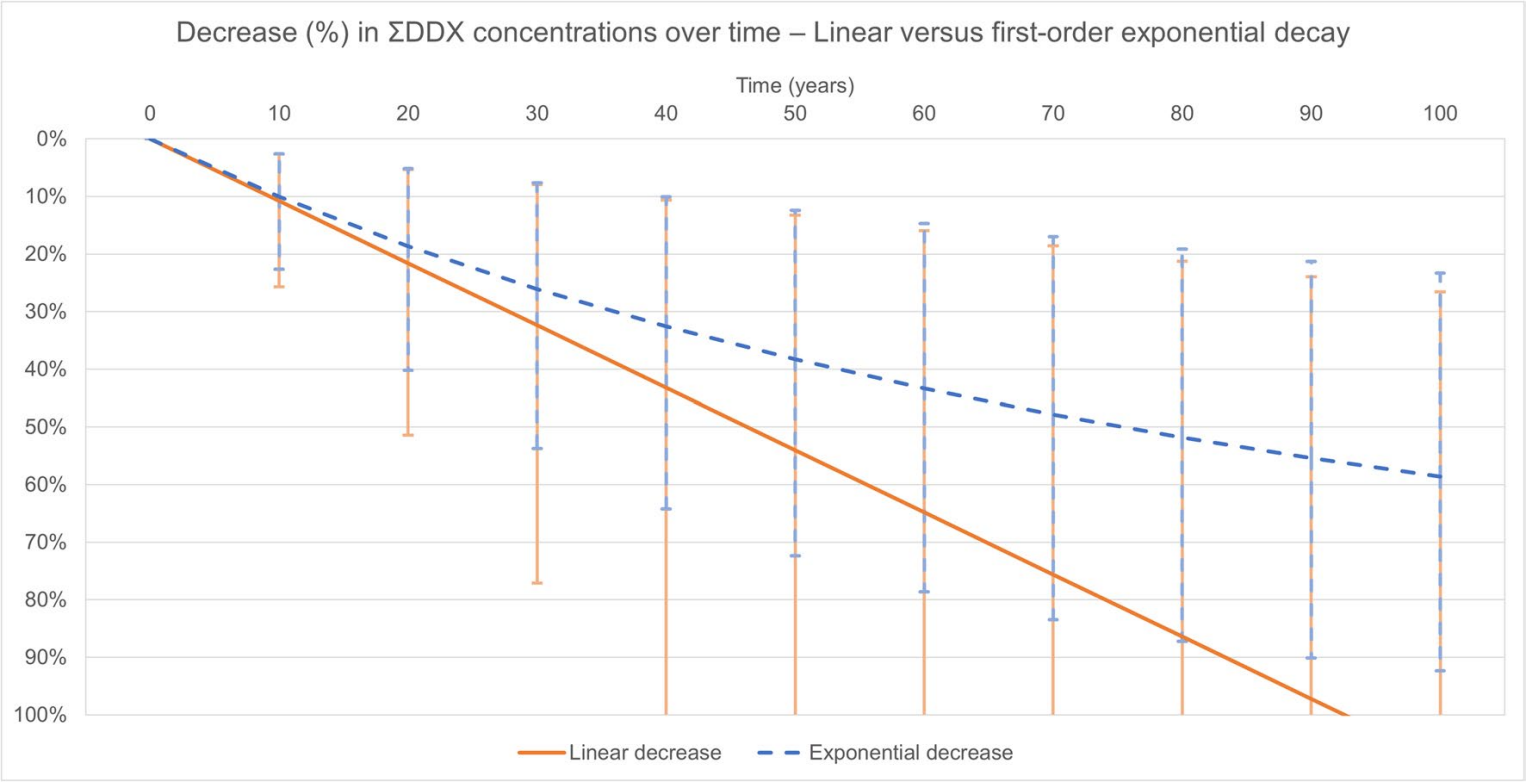
Steady-state linear extraction vs. first-order exponential decay extraction of ΣDDX from the soil by pumpkin (Cucurbita pepo ssp. pepo, cv. Howden), shown for literature data. Simulated results are mean values with the error bars representing the uncertainty interval [5th and 95th percentile], the solid orange line and error bars represents the linear model and the blue dashed line and error bars represents first-order exponential decay

The first-order exponential decay extraction model results in a much longer expected remediation time than the linear steady-state extraction model, which is shown in Fig. 1 for the literature dataset with a *C*_*soil,i*_ of 10 mg_DDX_/kg _dw_. The decrease in ΣDDX concentrations in soil is roughly similar during the first 20 years of phytoextraction but simulations with the first-order exponential model results in much less efficient removal soon thereafter as the ΣDDX pool diminishes. Also, the uncertainty intervals representing the 5th and 95th percentile of the probable percent decrease in ΣDDX concentration broaden over time indicating that there is greater uncertainty as to how the ΣDDX concentrations are expected to change in the long-term, especially for the linear analytical model.

### Time expectations and removal rates based on literature or site-specific data

The results from the probabilistic models, linear and the first-order exponential decay, and a comparative analysis between literature data and site-specific data for phytoextraction of ΣDDX with pumpkin are shown in Table 3 (and Figures S11, S13, S15, and S17 in SM). Large differences between input data for *BAF* and *BMP* result in a substantially higher mostly likely (*mode*) simulated removal rate (*k*) of 0.61% per year when using literature data compared to the site-specific data, with a much lower most likely simulated removal rate of only 0.013% per year. The models were applied to determine the expected time required to reduce the *C*_*soil,i*_ of ΣDDX of 10 mg_DDX_/kg _dw_ in the experimental plots to the regulatory soil guideline value in Sweden for less sensitive land uses of 1 mg_DDX_ /kg _dw_, which requires a likely unachievable 90% reduction in soil ΣDDX concentrations. When using literature data, the most likely remediation time is 47.9 years with an uncertainty interval for the 5th and 95th percentile of 35.3 and 340 years (hereafter shown in brackets as [5th; 95th]), respectively, using the linear extraction model, or 123 years [90.3; 870] when using the first-order exponential decay model. For the experimental data from Kolleberga, predicted remediation time is much longer: approximately 3 570 years [2 280;16 400] for the linear steady-state extraction model or 9 120 years [5 840; 42 000] when using the first-order exponential decay model.

**Table 3:** Results from simulations of phytoextraction using linear or first order exponential models – comparison between literature and site-specific data and estimated time required to reduce soil ΣDDX concentrations from 10 to 1 mg/kg _dw_ (≈90% reduction). Simulated results are the most likely value (mode), and the uncertainty interval [5th and 95th percentile] in brackets

	Removal rate, *k* (% per year)	Remediation time (years)	
Dataset		Linear	First-order
Literature	0.606% [0.267; 2.51]	47.9 [35.3; 340]	123 [90.3; 870]
Site-specific	0.0127% [5.43 E^−5^; 0.0392]	3 570 [2 280; 16 400]	9 120 [5 840; 42 000]

### Phytoextraction of specific DDT metabolites

The *BAF* of *C. pepo* can differ between the different DDT metabolites, which has proven to be valid in the Kolleberga field experiment (Table 4), as indicated by the different *BAF* and resulting mean simulated removal rates for the metabolites. Despite the removal rate being higher for *p,p’*-DDE, it would still most likely take approximately 1 400 years to reduce the initial concentration by 90% according to the linear analytical model. Even using the maximum BAF for *p,p’-*DDE reported in literature of 18 (Eevers et al. 2018), a 90% reduction of the initial *p,p’*-DDE concentration would still most likely require a time of 214 years by linear extraction, all other things being equal.

**Table 4:** Varying effectiveness for different DDX – site-specific data. Simulated results are generated using the linear model and reported as most likely values (mode), expected remediation time is estimated for a 90% reduction for each metabolite

	ΣDDX	*p,p’*-DDT	*o,p’*-DDT	p,p’-DDD	p,p’-DDE
Mean *BAF*_*stems*_	0.890	0.718	2.67	1.63	1.08
Mean *BAF*_*leaves*_	0.134	0.114	0.286	0.114	0.199
*C*_*soil,i*_ (mg/kg _dw_)	10	7.9	1.0	0.48	0.76
Extraction potential, *E* (mg/year)	0.743	0.436	0.236	0.0308	0.186
Removal rate, *k* (%/year)	0.0127%	0.0105%	0.0431%	0.0116%	0.0457%
Remediation time, *t*_*final*_ (years)	3 570	3 550	1 420	1 210	1 400

### Scenario analysis

#### Varying phytoextraction efficiency

The average *C*_*soil*_ in the experimental plots at Kolleberga is ca. 10 mg_DDX_/kg _dw_, which corresponds with a ‘high’ concentration of ΣDDX according to similar studies and may also exceed a ‘threshold’ at which the effectiveness of pumpkin for phytoextraction diminishes significantly (Denyes et al. 2016; Lunney et al. 2004; Paul et al. 2015). By testing different *C*_*soil,i*_ and adding an ‘efficiency gradient’ as a factor (%_eff_) to modify the extraction potential (*E*)*,* the models can account for the likely negative correlation of phytoextraction performance with increasing soil ΣDDX concentrations. As shown in Table 5, the efficiency factor greatly impacts the resulting removal rates and expected remediation times (using the linear model) between ‘high’, ‘moderate’ and ‘low’ levels of ΣDDX contamination, with a most likely simulated removal rate of 0.22% (214–555 years), 0.66% (63–127 years) or 1.0% (24.6–34.0 years), respectively.

**Table 5:** Estimated differences in time requirements to reduce different C_soil,i_ to target value of 1 mg_DDX_ kg _dw_ for a 1 m^2^ unit area –using literature data (input data in left box). Simulated results (right box) are most likely values (mode), and the uncertainty interval [5th and 95th percentile] in brackets

Efficiency gradient		Initial soil ΣDDX			Remediation time, *t*_*final*_ (years)	
ΣDDX Level	Efficiency factor (*%*_*eff*_)	*C*_soil,i_ (mg/kg _dw_)	*m*_*soil,i*_ (mg_DDX_)	Removal rate, *k* (% per year)	Linear	Exponential
High	33%	10	5250	0.219% [0.0900; 0.853]	214 [106; 1 000]	555 [276; 2 610]
	47%	9	4730	0.283% [0.126; 1.19]	150 [74.5; 704]	370 [184; 1 740]
	60%	8	4200	0.364% [0.162; 1.53]	115 [57.0; 539]	273 [136; 1 280]
	73%	7	3680	0.444% [0.198; 1.88]	92.0 [45.7; 432]	209 [104; 981]
	87%	6	3150	0.525% [0.234; 2.22]	75.7 [37.6; 356]	163 [80.8; 765]
Moderate	100%	5	2630	0.656% [0.270; 2.56]	63.0 [31.3; 296]	127 [62.9; 595]
	118%	4	2100	0.776% [0.311; 3.04]	48.2 [24.6;241]	89.1 [45.5; 445]
	136%	3	1580	0.895% [0.359; 3.51]	37.1 [19.0; 186]	61.2 [31.3; 308]
Low	155%	2	1050	1.01% [0.407; 3.98]	24.6 [12.6; 123]	34.0 [17.4; 170]

#### Simulations of increasing BAF and BMP

**Fig. 2:**
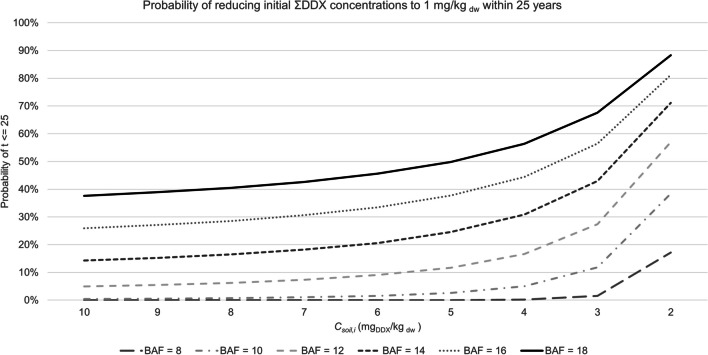
Simulations to determine what BAF is required to reduce initial C_soil_ to 1 mg kg^−1^ ΣDDX (corresponding to the soil guideline value for ‘less sensitive land use’ in Sweden) within 25 years for different initial C_soil_, calculated using literature data for linear analytical model – BMP_lit_,). The simulated values are most likely (mode) values

Results of simulations to determine the minimum required *BAF*_*stem*_ to reduce different *C*_*soil,i*_ to the Swedish soil guideline value of 1 mg_DDX_/kg _dw_ within 25 years (i.e., ‘a reasonable timeframe’ (Robinson et al. 2015)) are shown in Fig. 2. The simulations were run using literature data for *BAF*_*leaves*_, *BMP*_*stems*_ and *BMP*_*leaves*_ but testing different values for *BAF*_*stem*_: 8, 10, 12, 14, 16, and 18 in the linear phytoextraction model. The simulations show that there is a < 40% probability of reducing the ‘high’ *C*_*soil,i*_ of 10 mg_DDX_/kg _dw_ to the SGV within 25 years, even with the highest simulated *BAF*_*stem*_ value of 18. The probabilities remain consistent and increase slowly with decreasing ΣDDX concentrations until reaching the ‘moderate’ *C*_*soil,i*_ of 5 mg_DDX_/kg _dw_ where a *BAF*_*stem*_ of 18 and 16 have an approximately 50% and 38% probability to achieve a 90% reduction within 25 years, respectively. The probabilities increase more sharply approaching a ‘low’ *C*_*soil,i*_, and a *BAF*_*stem*_ of 18, 16, 14, and 12 results in a > 50% of achieving the 90% reduction target within 25 years for an *C*_*soil,i*_ of 2 mg_DDX_/kg _dw_. The lowest tested *BAF*_*stem*_ of 8 and 10 have a < 10% probability of achieving the 90% reduction target within 25 years for all concentrations above 4 mg_DDX_/kg _dw_ and do not exceed a 20% or 40% probability, respectively, for even the lowest *C*_*soil,i*_ of 2 mg_DDX_/kg _dw_.

Even the scenario of ‘optimised’ growth of pumpkin using site-specific data for *BAF*_*stem*_ and *BAF*_*leaves*_ but literature derived *BMP* (*BMP*_*lit*_) does not indicate that phytoextraction would be feasible. The most likely (*mode*) time required to achieve a 90% reduction of ΣDDX by linear extraction would be approximately 380 [288; 1 690] years or 986 [737; 4 320] years for first-order exponential decay though the most likely simulated removal rate improves substantially from the previously reported 0.0127% in “Time expectations and removal rates based on literature or site-specific data” section to approximately 0.178% of soil ΣDDX removed per year.

## Discussion

### Model evaluation – literature versus site-specific datasets

Large differences in *BAF* and *BMP* derived from literature and the site-specific dataset from Kolleberga (Table 3; Table S2 & S3) result in a substantial gap between the corresponding time estimates, which indicates the difficulty in generalising results between different studies and sites. The differences may be due to site-specific conditions (e.g., soil type, climate, soil physical and chemical parameters) that result in a suboptimal soil environment for pumpkin growth and/or a lower DDX uptake at Kolleberga. Pumpkins in general require well-drained sandy soils with high contents of organic matter and nutrients, sunlight, air temperatures of 24–35°C, neutral pH, regular irrigation and fertilization for optimal growth of the harvestable biomass (Johnny’s Selected Seeds 2018; UMass Extension Center for Agriculture 2012). Although the experiments in Canada by Paul et al. (2015) and Denyes et al. (2016) are similar (sandy soils, climate), Kolleberga has a lower TOC (1.6%) and likely insufficient nutrient availability since the Kolleberga experiment was not designed to optimise pumpkin growth.

Compared to literature data, the site-specific *BMP*_*stem*_ is tenfold lower, and the *BMP*_*leaves*_ a little less than half. The site-specific *BMP* was adjusted in the “optimal” scenario to partially correct for the poor pumpkin growth in the 2nd season, which introduces significant uncertainty in the values. However, the range of adjusted values for the high DDT-contaminated Kolleberga site are still much lower compared to comparable literature values: a mean stem dry weight of 19.6 g for 4 plants/m^2^ compared to 88.4 g for only 1 plant/m^2^ (Paul et al. 2015). Improving the *BMP* and reducing the uncertainties in the estimate could greatly reduce resulting time estimates, as shown in the sensitivity analysis (Figures S12, S14, S16, S18). However, in general, there are still uncertainties in predicting *BMP* since the ΣDDX concentration can affect plant growth and it may also decrease over time due to e.g., nutrient depletion after several harvests, or due to variable environmental factors causing unforeseen complications such as pests, disease or climatic conditions like drought (Van Nevel et al. 2007).

In terms of DDX uptake, studies reported values of *BAF*_*stem*_ specifically for *C. pepo* cv. Howden of 0.8–4.5 for ΣDDX (Denyes et al. 2016; Lunney et al. 2010, 2004; Paul et al. 2015; Whitfield Åslund et al. 2010) and even 7.2 for *p,p’-*DDE (White 2002). These are much higher than the mean *BAF*_*stem*_ of 0.89 in the Kolleberga field experiment, where a BAF < 1 indicates ineffective accumulation. A likely explanation for the comparatively low *BAF* and *BMP* observed at Kolleberga is that the ΣDDX concentrations (mean *C*_*soil*_ ~ 10 mg_DDX_/kg _dw_) exceed a threshold below which pumpkin can effectively phytoextract DDX. Indeed, several studies have shown that plants used for phytoextraction for different types of contaminants (e.g., metals or POPs) are most effective at removing moderate amounts of contaminants but have a ‘threshold’ at which phytoextraction potential peaks then decreases due to toxic effects on plant physiology and growth and plant avoidance strategies (Audet and Charest 2007; Dehghani et al. 2021; Denyes et al. 2016; Paul et al. 2015). For *C. pepo* cv. Howden, such thresholds have been reported at ca. 10 mg/kg _dw_ (Denyes et al. 2016) or 5 mg/kg _dw_ (Paul et al. 2015). For instance, Paul et al. (2015) showed that the phytoextraction efficiency was greatest in the ‘low’ ΣDDT contaminated soil (ca. 0.291 mg/kg _dw_) with a *BAF*_*stem*_ of 4.5, although the overall extraction potential for total removal was greatest in the ‘moderately’ contaminated soil (ca. 5.1 mg/kg _dw_) with a *BAF*_*stem*_ of 2.4, was greatly diminished at the ‘high’ concentration (ca. 10.2 mg/kg _dw_) with a *BAF*_*stem*_ of 0.8, which is consistent with the Kolleberga field experiment. Overall, the experimental results at Kolleberga are comparable to Paul et al. (2015) who achieved an average removal rate of approximately 0.014% per year for the ‘high’ ΣDDT contaminated site.

### Implications for phytoextraction at Kolleberga

The exponential model, which accounts for a decreasing contaminant pool over time, resulted in most likely time estimates of 123 years using literature data or 9 120 years using site-specific data to reduce *C*_*soil*_ by 90% and is far beyond a ‘reasonable timeframe’ for phytoextraction of less than 10 or 25 years (Robinson et al. 2015; Vangronsveld et al. 2009). Thus, phytoextraction with pumpkin is not likely to be feasible at Kolleberga, or indeed similar sites, where the ΣDDX concentrations are above the threshold range of 5 – 10 mg_DDX_/kg_soil_ _dw_ (Denyes et al. 2016; Paul et al. 2015) and the target value is based on a total concentration of 1 mg_DDX_/kg_soil_ _dw_. As shown in the scenario “A” analysis, where different *C*_*soil,i*_ and efficiency factors were used to increase or decrease the extraction potential, *E* (Table 5), the predicted remediation time was reduced from 555 to 127 years at a *C*_*soil,i*_ of 10 or 5 mg_DDX_/kg _dw_, respectively, according to the first-order exponential decay model. The predicted timeframe is much shorter approaching ‘low’ DDX concentrations: as short as 34.0 years at 2 mg_DDX_/kg_soil_ _dw_. The applied efficiency gradient is however a simplification that likely overestimates the time predictions since the efficiency would be expected to change over time and possibly improve at lower concentrations although the available DDX pool would also diminish as *C*_*soil*_ decreases over time.

Much research on phytoextraction of ΣDDX has focused on the uptake on *p,p’-*DDE in particular due to its tendency to bioaccumulate in human fatty tissue (Antignac et al. 2023; Beard 2006) and usually being the most abundant and persistent degradation product of DDT at many sites (e.g. Eevers et al. 2018; Kelsey and White 2005; Wang et al. 2004; White 2002, 2001; White et al. 2006a, 2005b). However, at Kolleberga, *p,p’-*DDT (77%) and *o,p’-*DDT (9%) are present in greater concentrations with a smaller proportion of *p,p’*-DDE (8%) and *p,p’*-DDD (4%). Further, the site-specific data showed a difference in *BAF* for different metabolites (*BAF*_*stem*_ lowest for *p,p’*-DDT, but > 1 for *o,p’-*DDT, *p,p’-*DDE and *p,p’-*DDD, Table 4), indicating a potential for phytoextraction for certain metabolites but not the one which makes up the greatest proportion of the ΣDDX at Kolleberga. An aggregated *BAF* for ΣDDX may indeed not be truly representative of the total uptake and can differ substantially between sites with different ΣDDX compositions.

The simulations with different values for *BAF* and *BMP* aimed to determine the necessary effectiveness for phytoextraction at Kolleberga with pumpkin to be feasible (Table 5, Fig. 2). To improve the prospects of phytoextraction of ΣDDX at Kolleberga, the removal rate would need to be greatly increased. This could be done through enhancing pumpkin’s *BAF*, which has been done successfully by using biosurfactants such as *Pseudomonas* spp. (Wang et al. 2017; White et al. 2006b), mycorrhizal fungi (White et al. 2006a, b; Whitfield Åslund et al. 2010), bioaugmentation with endophytic bacteria (Eevers et al. 2018), earthworms (Kelsey and White 2005), and chemical surfactants or organic acids (White et al. 2007, 2003; Whitfield Åslund et al. 2010). The maximum tested *BAF*_*stem*_ in the models was 18 for ΣDDX and is likely unattainable; however, Eevers et al. (2018) achieved a *BAF*_*stem*_ of 18 for the metabolite *p,p’*-DDE (*C*_*soil*_ of ca. 0.15 mg/kg _dw_) in their study by inoculating zucchini (*C. pepo* ssp. *pepo* cv. Raven) with a consortium of DDE-degrading endophytes derived from zucchini, which the authors suggest improves phytoextraction’s feasibility by improving plant growth and overall *p,p’-*DDE removal by promoting biological degradation. Similarly, various agronomic practices have been tested to improve the *BMP* of *C. pepo* for phytoextraction of DDT (Denyes et al. 2016; Lunney et al. 2010; White et al. 2005a; Whitfield Åslund et al. 2010). Using high biomass producing species and further improving the amount of produced biomass through the use of organic soil amendments, microbial amendments such as mycorrhizal fungi, and other agronomic practices is a widely accepted strategy to improve *BMP* and thus the effectiveness of phytoextraction (Kidd et al. 2015; Mench et al. 2010; Vangronsveld et al. 2009).

The bioavailability of contaminants is determined by soil environmental conditions and complex interactions with pH, soil organic matter content, water availability, soil biota, carbonates and clay content as well as aging of contaminants (Antoniadis et al. 2017; Canales-Pastrana and Paredes 2013; Kumpiene et al. 2017; Petruzzelli et al. 2020; Sauvé et al. 2000). Many of the beforementioned studies using cv. Howden for phytoextraction are tested for field-weathered or aged DDT, which is also true for Kolleberga. Aging effects have been reported to lead to strong sorption of hydrophobic organic compounds, such as PAHs and DDT, to different kinds of natural organic matter in the soil over time (Wang et al. 2012) thereby greatly reducing their bioavailability. Tang et al. (1999) showed that the amount assimilated in earthworm tissue was considerably lower in soils with DDT aged for 49 years compared to soils with freshly added DDT; and Morrison et al. (2000) reported either 30%, 12%, 34% or 20% of the total DDT, DDE, DDD and ΣDDT, respectively, was detected in earthworms after exposure to a field soil treated with DDT 49 years earlier. Smith et al. (2012) determined that the relative bioavailability of ΣDDT, as measured according to accumulation in different organ tissues in mice, did not exceed 25%. Passive sampling methods such as polyoxymethylene (POM) can also provide accurate measures of changes in bioavailability (e.g., as a result of treatment) by using porewater concentrations as a proxy for bioavailability to support risk assessments, though they may overestimate the expected uptake into plants (Denyes et al. 2016). Wang et al. (2018) tested Tenax desorption and the isotope dilution method (IDM) to measure the bioavailable fraction of DDX in a historically contaminated soil, which ranged from 40.6–80.6% that varied for different metabolites and methods. In general, bioavailability of ΣDDX for plants plays an important role in phytoextraction effectiveness since only a fraction of the total contaminant concentration is potentially available for uptake into plants. The *BAF* used here is a direct measure of the uptake of DDX into pumpkin, but it would be beneficial to complement with a more accurate estimation of the actual bioavailable fraction of DDX.

### Application, limitations, and uncertainties of the probabilistic phytoextraction model

Although the analytical probabilistic models are simplified, they can be useful to provide an initial estimation of the remediation time required for phytoextraction at a particular site accounting for several uncertainties, and could complement more generalised approximations such as those proposed in Drenning et al. (2022). Indeed, a simplified approach based on empirical data from either literature or relatively inexpensive pilot experiments could be applicable for communicating with decision-makers and other stakeholders to clarify uncertainties in time expectations for a particular site and the perceived uncontrollability of phytoextraction, which are always important factors determining feasibility. The methodology followed here to model phytoextraction for ΣDDX using pumpkin can also be used for other plants and contaminants, e.g., for phytoextraction of metal(loid)s using willow (*Salix* sp.) for which there are many studies to gather empirical data. Important outputs from the model to inform stakeholders include the most likely time required, the projected minimum and maximum time required for remediation (as an uncertainty interval using the 5th and 95th percentiles), the minimum *BAF* and *BMP* needed, as well as the associated uncertainties. The contaminant extraction potential, *E*, and removal rate, *k*, are useful to assess phytoextraction feasibility, but are likely to decrease over time as contaminant concentration and the available pool of contaminants decreases (Herzig et al. 2014; Mo et al. 2008; Neaman et al. 2020; Robinson et al. 2015; Santa-Cruz et al. 2022; Van Nevel et al. 2007; Vangronsveld et al. 2009).

Many studies have reported the oversimplification of linear analytical models that assume that biomass production, accumulation and bioavailability of contaminants do not change over time, e.g., (Manzoni et al. 2011; Neaman et al. 2020; Shi et al. 2023; Thijs et al. 2018). Nevertheless, it can still be useful and provide an indication of which scenarios are not suitable for phytoextraction (Robinson et al. 2015, 2006; Thijs et al. 2018). The first-order exponential decay model may better reflect changing conditions by incorporating, to some extent, temporal heterogeneity and a more complicated soil chemistry (with various sorption, retention and leaching processes) that model decreases in the available contaminant pool over time (Herzig et al. 2014; Manzoni et al. 2011; Robinson et al. 2015, 2009, 2006, 2003b; Van Nevel et al. 2007; Vangronsveld et al. 2009). Neither analytical model explicitly considers bioavailability and spatial heterogeneity, and some authors have pointed out that the empirical uptake factors used in such analytical models can overestimate the effectiveness of remediation (Raguž et al. 2013; Santa-Cruz et al. 2022).

Temporal and spatial heterogeneity greatly impact phytoextraction and can be a major reason why effectiveness is overestimated (Keller 2005; Keller et al. 2003; Robinson et al. 2015, 2006; Van Nevel et al. 2007; Vangronsveld et al. 2009). Spatial heterogeneity, of contaminants, nutrients, and water in soil, as well as heterogeneity of plant roots has a large, but uncertain effect on phytoextraction effectiveness (Robinson et al. 2015). Accounting for temporal heterogeneity and bioavailability, including to what extent the labile contaminant pools are depleted and recharged over time or completely immobilised and unavailable to plants require much more complex, mechanistic models. In this approach, the bioavailability is considered to be embedded in the empirical data to calculate *BAF* and the probability distributions are assumed to account for the variability in effectiveness that may be caused by changing contaminant availability, heterogeneity and other aspects that may negatively impact phytoextraction.

For organic contaminants such as DDT, the potential role that biological degradation would play in decreasing concentrations over time alongside, or instead of, phytoextraction is important to consider but is not included in the models. Indeed, Mo et al. (2008) suggest that phytoextraction may play a limited role in the removal of ΣDDX from soils (< 1%) and DDT metabolism or biodegradation may instead be the main mechanism for removal.

### Phytoextraction feasibility

Given phytoextraction’s inherent limitations and inefficiencies, it is reasonable to ask what is possible to achieve, and for which situations would it be feasible? As demonstrated in this study, reducing the ΣDDX concentrations to an ‘acceptable level’ requiring a 90% reduction in the initial total soil concentration would most likely take multiple decades, if not centuries, even under optimal growing conditions. In addition, the pool of readily available ΣDDX at Kolleberga and similar sites, is likely to decrease over time due to aging effects and make it "permanently" inaccessible to soil organisms or plant roots. Thus, it may be impossible for plants to remove the entire total concentration of ΣDDX from the soil and achieve a 90% reduction target. However, from a risk perspective, this may be beneficial, especially if the main risks are related to the soil ecosystem and to bioaccumulation in the food chain. Thus, the bioavailability of the contaminant is an important factor to consider, not least for the risk assessment.

Due to the excessive time requirements to achieve reduction targets, many authors consider phytoextraction to be infeasible in most cases, especially if national regulation is based on total soil contaminant concentrations instead of bioavailable concentrations (Dickinson et al. 2009; Mertens et al. 2005; Neaman et al. 2020; Robinson et al. 2015; Santa-Cruz et al. 2022; Van Nevel et al. 2007). The long timeframes predicted at Kolleberga align with many recent studies that report that phytoextraction may only be feasible at lower contaminant concentrations. Alternative strategies should thus be considered for Kolleberga, such as combining with other technologies as part of a ‘treatment chain’, ‘soil polishing’ (reducing marginally elevated concentrations to acceptable levels), or ‘bioavailable contaminant stripping’ (reducing the soluble, bioavailable fraction of contaminants thereby reducing environmental risk), which can shorten remediation times from decades to just a few years (Delplanque et al. 2013; Dickinson et al. 2009; Gerhardt et al. 2017; Herzig et al. 2014; Mench et al. 2010; Neaman et al. 2020; Robinson et al. 2015, 2009, 2006; Van Nevel et al. 2007; Vangronsveld et al. 2009). There are, however, still obstacles and uncertainties regarding replenishment of bioavailable pools and such alternative risk-based land management strategies may not be acceptable to regulatory agencies (Neaman et al. 2020; Santa-Cruz et al. 2022; Thijs et al. 2018).

## Conclusion

Despite their simplicity, the presented models can be useful for communicating expectations and evaluating the initial feasibility of phytoextraction based on literature data of *BAF* and *BMP*, and determining for which situations phytoextraction may be applicable for a particular site, though there may be difficulties in generalising results and comparing between studies and sites. Empirical data from field studies can be used to generate probability distributions for *BAF* and *BMP* at a particular site and decrease the uncertainty of the output compared to literature data. The analytical probabilistic models can be used to calculate the phytoextraction potential and removal rate, provide estimates on the most likely time requirements, the minimum and maximum time uncertainty intervals, as well as the minimum *BAF* and *BMP* needed to achieve acceptable contaminant concentration levels in the soil within a reasonable timeframe of 25 years while accounting for uncertainties. To reduce the initial total concentration of 10 mg_DDX_/kg_soil_ _dw_ in the field experiment at Kolleberga by 90% to the Swedish soil guideline value of 1 mg_DDX_/kg_soil_ _dw_, results from the linear model indicate a most likely (mode) time required ranging from 47.9 to 3 570 years while the first-order exponential model indicates 123 to 9 120 years are required using literature or site-specific data, respectively. Phytoextraction is thus infeasible for the fields at Kolleberga, with ΣDDX concentrations ranging from 5–15 mg/kg _dw_, within a reasonable time frame due to low uptake into pumpkin aboveground biomass and low biomass production. Nevertheless, the effectiveness can be improved with bioinoculants and at lower initial soil ΣDDX concentrations where a strategy of ‘soil polishing’, ‘bioavailable contaminant stripping’, or as part of a ‘treatment chain’ could potentially be feasible to manage areas at Kolleberga with ΣDDX concentrations lower than 5 mg/kg _dw_. However, for such strategy to be feasible, the bioavailability and the plant available pool of ΣDDX needs to be investigated and considered in the risk assessment and the remediation target instead of only total ΣDDX soil concentrations. Alternative risk management strategies such as combining phytoextraction with stimulated biological degradation of ΣDDX or stabilisation using amendments such as biochar could be more promising for Kolleberga and should also be investigated.

### Supplementary Information

Below is the link to the electronic supplementary material.

Supplementary file1 (DOCX 2991 KB)

## Data Availability

Data is available upon request.
