# Supplementary material for: Development of simplified probabilistic models for predicting phytoextraction timeframes of soil contaminants: demonstration at the DDX-contaminated Kolleberga tree nursery in Sweden
Source: Environ Sci Pollut Res Int. 2024 Jun 5;31(28):40925–40. doi: 10.1007/s11356-024-33858-x (PMC11189973; doi:10.1007/s11356-024-33858-x)
Supplement: Supplementary file 1 — Supplementary file1 (DOCX 2991 KB) [file 11356_2024_33858_MOESM1_ESM.docx]

ASupplementary Material

Probabilistic models to estimate the time required for phytoextraction

Paul Drenning^1*^, Anja Enell^2^, Dan Berggren Kleja^2,3^, Yevheniya Volchko^1^, Jenny Norrman^1^

^1^Department of Architecture and Civil Engineering, Chalmers University of Technology, SE 412-96

^2^ Swedish Geotechnical Institute (SGI), SE-581 93 Linköping, Sweden

^3^ Department of Soil and Environment, Swedish University of Agricultural Sciences (SLU), Box 7014, SE-750 07 Uppsala, Sweden

Table of Contents

[1 Site and experiment description 2](#_Toc159572393)

[1.1 Experimental set-up 2](#_Toc159572394)

[1.2 Soil DDX 4](#_Toc159572395)

[2 Input variables and associated probability distributions 5](#_Toc159572396)

[2.1 Experimental data 7](#_Toc159572397)

[2.1.1 Pumpkin BAF 8](#_Toc159572398)

[2.1.2 Pumpkin BMP 9](#_Toc159572399)

[2.2 Literature data 10](#_Toc159572400)

[2.2.1 Pumpkin BAF 10](#_Toc159572401)

[2.2.2 Pumpkin BMP 11](#_Toc159572402)

[3 Results and sensitivity analysis 12](#_Toc159572403)

[3.1 Site-specific data 13](#_Toc159572404)

[3.2 Literature data 15](#_Toc159572405)

# Site and experiment description

## Experimental set-up

This study is part of a 3-year field experiment at the Kolleberga tree nursery in Ljungbyhed (Southern Sweden) where pumpkin (*Cucurbita pepo* ssp. *pepo,* cv. Howden) was evaluated for its phytoextraction potential. The experimental set-up and overall experiment are described in more detail below.

A pilot-scale field experiment was established at the Kolleberga tree nursery site according to a randomized block design of test plots after thoroughly homogenizing the soil in a pile and mixing half of the volume of soil with biochar. Treatments consisted of four different types of plants, with or without biochar addition to the soil, and were established in triplicate (in total 24 plots). The four different plants are aimed at different phytoremediation strategies: 1) phytoextraction (pumpkin), 2) aided phytostabilisation (willow, grass-mixture) with biochar expected to facilitate immobilisation, and 3) phyto/rhizodegradation (nitrogen-fixing plants, such as clover and alfalfa) Figure S2.

The following steps were taken to establish the field experiment (Figure S1):

1. A transect of 50x5m was excavated to ca. 35cm depth below ground level (depth of contamination/plough depth) and moved to a soil pile.
2. The soil pile was mixed to homogenize the soil and half was mixed with biochar at a 3% w/w ratio.
3. 24 experimental plots of 2x2m and 35cm depth were dug in the trial area and a fiber cloth was put into the bottom to contain the soil and roots within the soil volume.
4. The soil was randomly distributed into the plots – half with and half without biochar – corresponding to a randomized block design, i.e., in triplicate but separated into 3 blocks that contained each of the 8 treatments. Four different plants mixes were established in the 24 plots, including pumpkin, grass mix, legume mix, and willow.
5. The remaining soil in the pile and from digging the experimental plots was put back into the excavation area to restore the excavation.


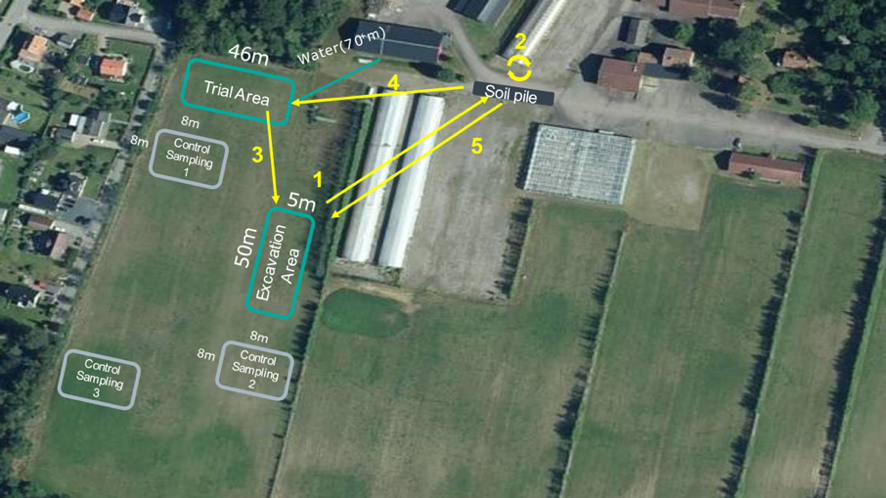


Figure S1. Steps followed to set up the field experiment.


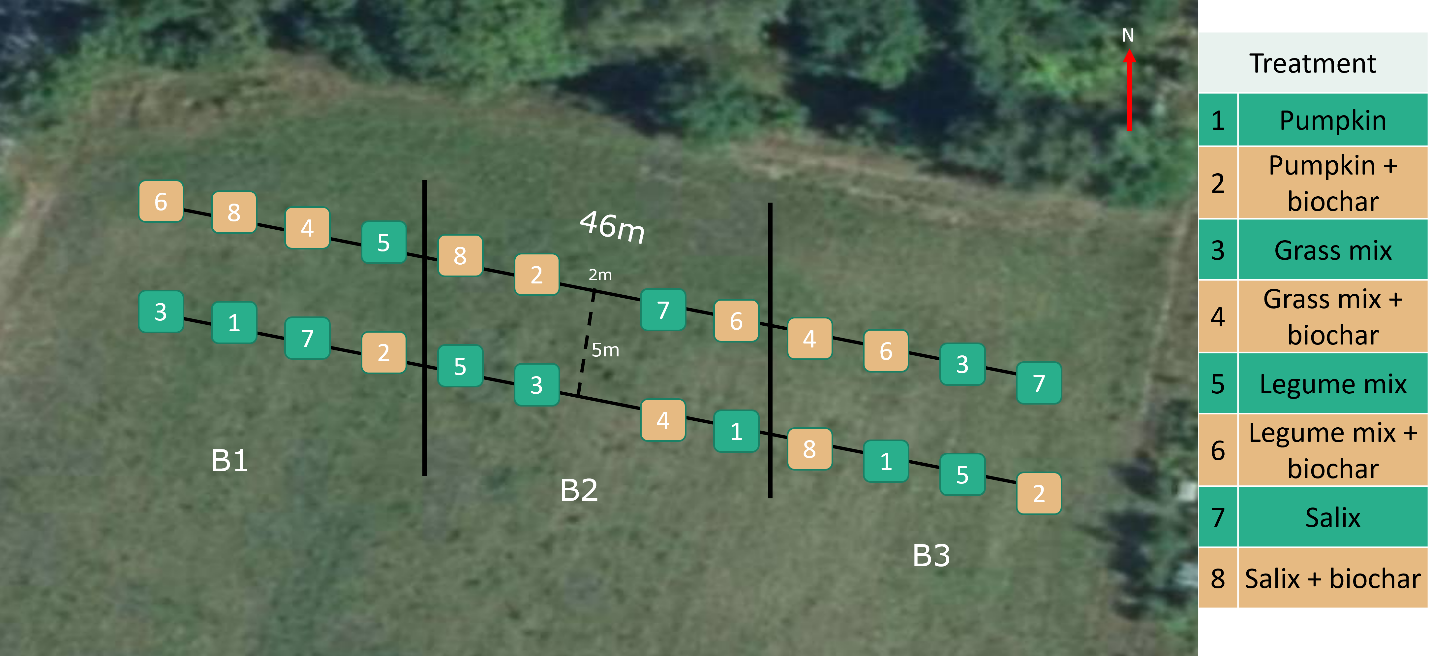


Figure S2. Overview map of the experimental area and treatments in a block design. Treatment numbers 1-8 correspond to the table, orange boxes are where biochar was mixed into the soil.

## Soil DDX

Table S1 summarizes the site-specific soil ΣDDX concentrations used in the phytoextraction model.

Table S1. Mean ΣDDX concentrations in the field soil at Kolleberga (n=6). Final concentrations calculated based on a 90% reduction to achieve the acceptable level for ‘sensitive land use’ according to Swedish legislation.

| **Site data** | ΣDDX | *p,p’-*DDT | *o,p’-*DDT | *p,p’-*DDD | *p,p’-*DDE, |
| --- | --- | --- | --- | --- | --- |
| Cont. concentration (mg/kg _dw_) - *Initial* | 10.0 | 7.93 | 1.04 | 0.505 | 0.773 |
| Cont. concentration (mg/kg _dw_) - *Final* | 1 | 0.793 | 0.104 | 0.0505 | 0.0773 |
|  |  |  |  |  |  |
| **DDX, m_i_ (**mg) **-** *Initial* | 5250 | 4170 | 548 | 265 | 406 |
| **DDX, m_f_ (**mg) **-** *Final* | 525 | 417 | 54.8 | 26.5 | 40.6 |

# Input variables and associated probability distributions

Probability distributions were assigned depending on the type of input variable and available data. Beta-PERT distributions were used for variables for which minimum, maximum and most likely values could be derived from literature. Most of the simulations will generate values near the most likely value (mode) of the distribution. Normal distributions are used for all experimental data. The values used for each input variable in the model with their associated probability distributions are summarized in Table S2 and shown for each variables in Figures Figure S3-
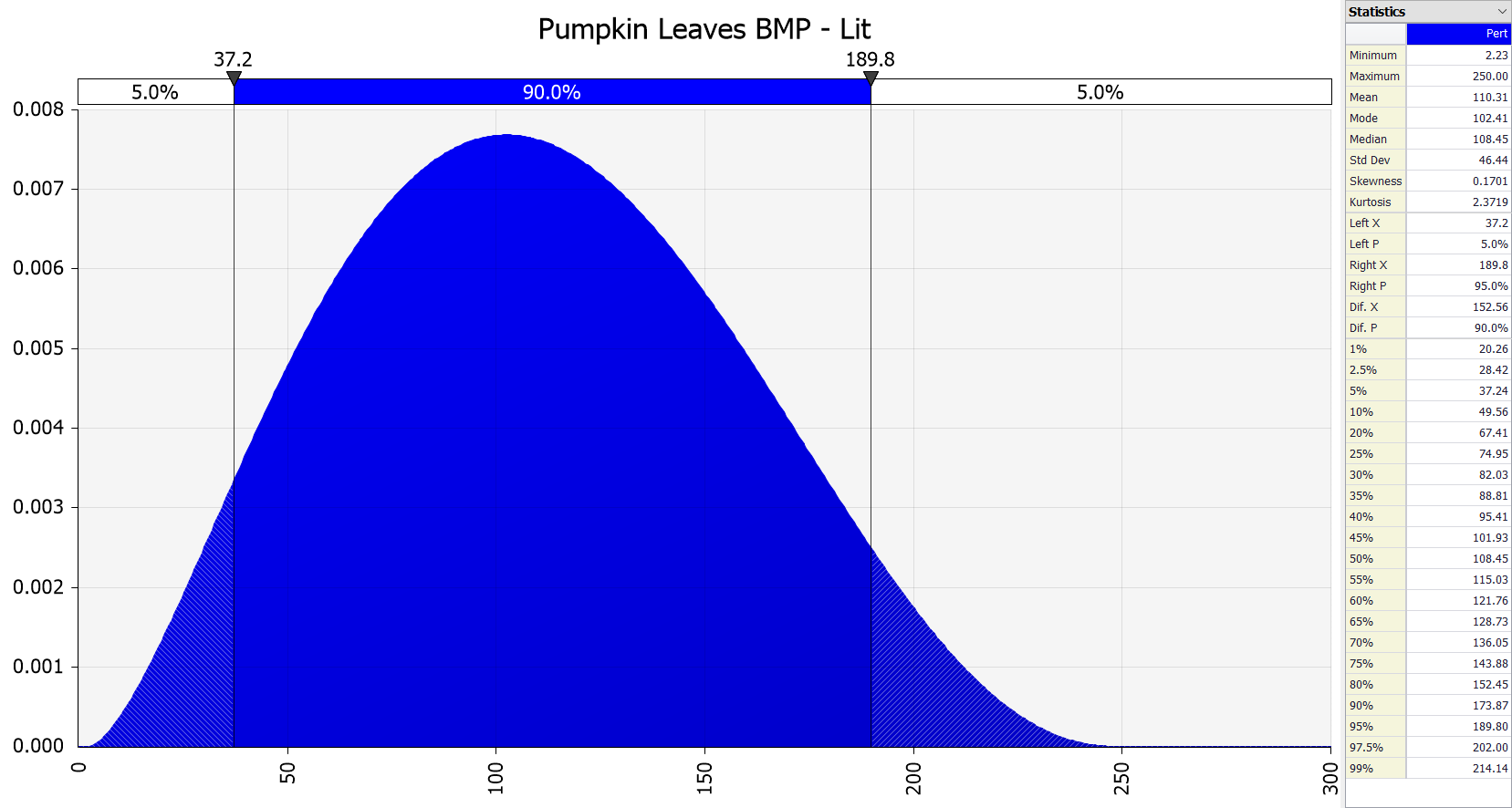
Figure S10.

Table S2. Input variables, probability distributions and associated parameters used in the uncertainty analysis. Dist. = probability distributions; Min = minimum value; Max = maximum value; M. likely = most likely value; µ = mean; σ = standard deviation.

| Input variable | Dist | Parameters | Reference/comment |
| --- | --- | --- | --- |
| *Literature* | | | |
| *BAF_stem_* (ΣDDX) | Beta-PERT | Min: 0.8; Max: 22; M. likely: 2.9 | Paul et al. (2015); White (2002); Lunney et al. (2004; 2010); Whitfield Åslund et al. (2010); Denyes et al. (2016); White (2009); Wang et al. (2004) |
| *BAF_leaves_* (ΣDDX) | Beta-PERT | Min: 0.079; Max: 1.24; M. likely: 0.32 |  |
| *BMP_stem_* (g _dw_ per plant^*^) | Beta-PERT | Min: 88.4; Max: 550; M. likely: 200 | Paul et al. (2015); White (2002); White (2009); Wang et al. (2004) |
| *BMP_leaves_* (g _dw_ per plant^*^) | Beta-PERT | Min: 2.23; Max: 250; M. likely: 102 |  |
| *Site-specific (Kolleberga tree nursery)* | | | |
| *BAF_stem_* (ΣDDX) | Normal | µ: 0.89; σ: 0.46 |  |
| *BAF_leaves_* (ΣDDX) | Normal | µ: 0.13; σ: 0.08 |  |
| *BMP_stem_* (g _dw_ per plant^*^) | Normal | µ: 19.6; σ: 10.4 |  |
| *BMP_leaves_* (g _dw_ per plant^*^) | Normal | µ: 47.5; σ: 20.2 |  |

^*^*^BMP calculated based on 4 plants per square meter.^*

Table S3. Summary table of pumpkin bioaccumulation factors (BAF) for stems and roots and translocation factors (TF) for the first two years of the field experiment (Y1 & Y2).

| **Pumpkin without biochar** | | ΣDDX | DDT, o,p'- | DDT, p,p'- | DDD, o,p'- | DDD, p,p'- | DDE, o,p'- | DDE, p,p'- |
| --- | --- | --- | --- | --- | --- | --- | --- | --- |
| BAF Stems | Stems Y1 | 0,69 | 2,72 | 0,44 | 0,45 | 0,24 | 1,53 | 0,86 |
|  | Stems Y2^*^ | 0,28 | 0,96 | 0,20 | 0,31 | 0,13 | 1,20 | 0,37 |
| BAF Roots | Roots - Y1 | 1,69 | 8,54 | 0,76 | 0,75 | 0,24 | 4,12 | 3,53 |
|  | Roots - Y2^*^ | 2,62 | 8,23 | 1,75 | 2,42 | 1,62 | 6,88 | 4,43 |
| TF | Stems:Roots – Y1 | 0,41 | 0,32 | 0,58 | 0,59 | 0,97 | 0,37 | 0,24 |
|  | Stems:Roots – Y2^*^ | 0,11 | 0,12 | 0,11 | 0,13 | 0,08 | 0,17 | 0,08 |

^*Results presented for Y2 are the raw data without adjusting for Spanish slug infestation (described in section 2.1).^

## Experimental data

Empirical data from the first two years of the field experiment using pumpkin (*Cucurbita pepo* ssp. *pepo*) for phytoextraction of ΣDDX were used to calculate ranges and mean values for the two main variables: *BAF* and *BMP*. Due to a Spanish slug infestation consuming the second year’s harvest, the raw data was adjusted to extrapolate the potential uptake of ΣDDX in the second year if it was able to complete its growth cycle. The potential stem concentration in the second year was calculated by multiplying the root concentration of ΣDDX (mean uptake of 27 900 µg kg^-1^) and metabolites in the second year by the translocation factor (TF), which was derived from the first year’s harvest (mean stem ΣDDX TF = 0.41). With this adjusted data, an average stem *BAF* and standard deviation could be calculated to create a normal probability distribution.

In addition, since there was only negligible stem growth of pumpkin in the second year, the distribution of potential harvestable *BMP* was created using the mean *BMP* and standard deviation from the first year, with and without biochar addition.

### Pumpkin BAF


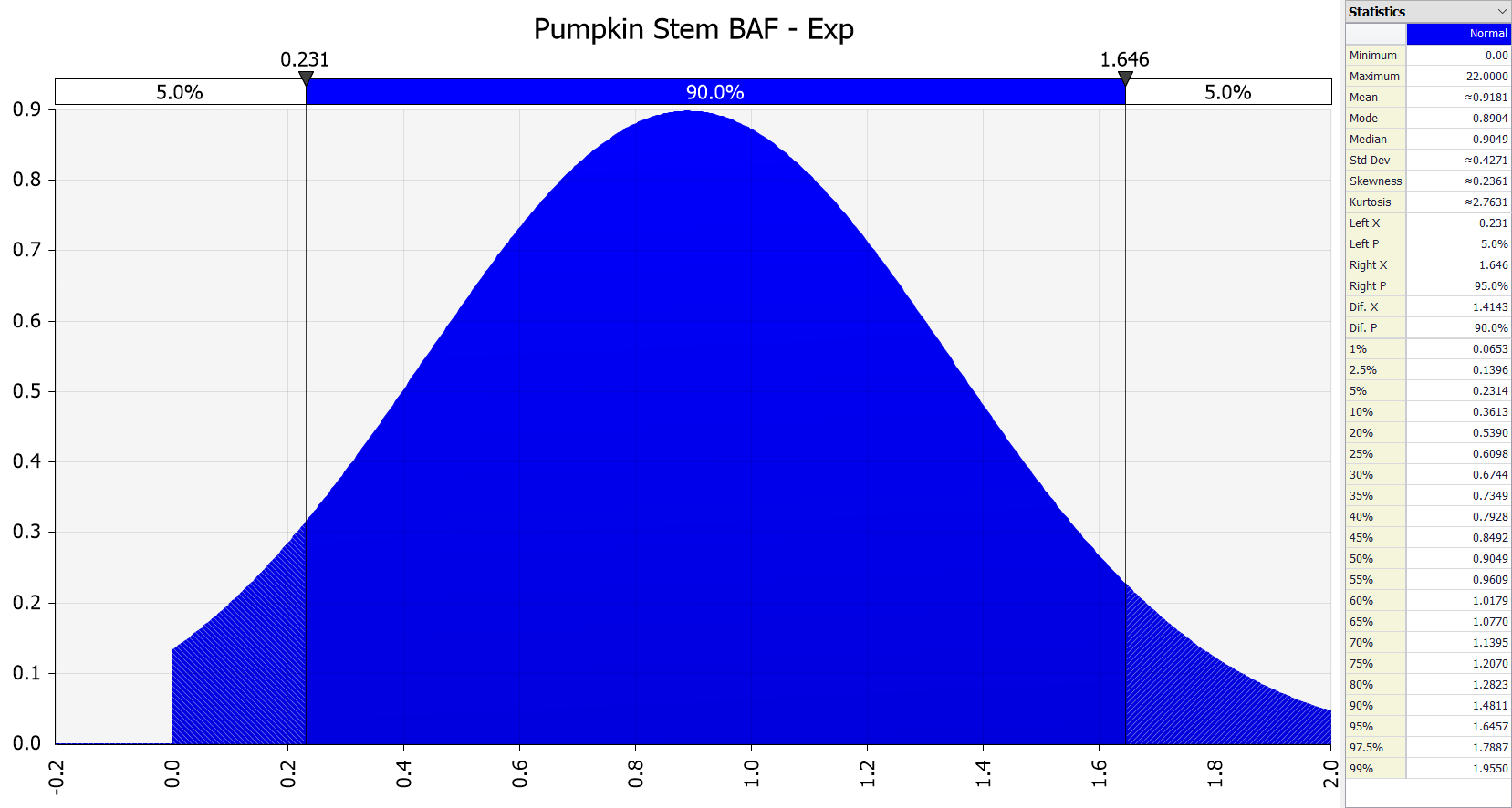


Figure S3. Probability distribution function for BAF_stem_ based on site-specific data.


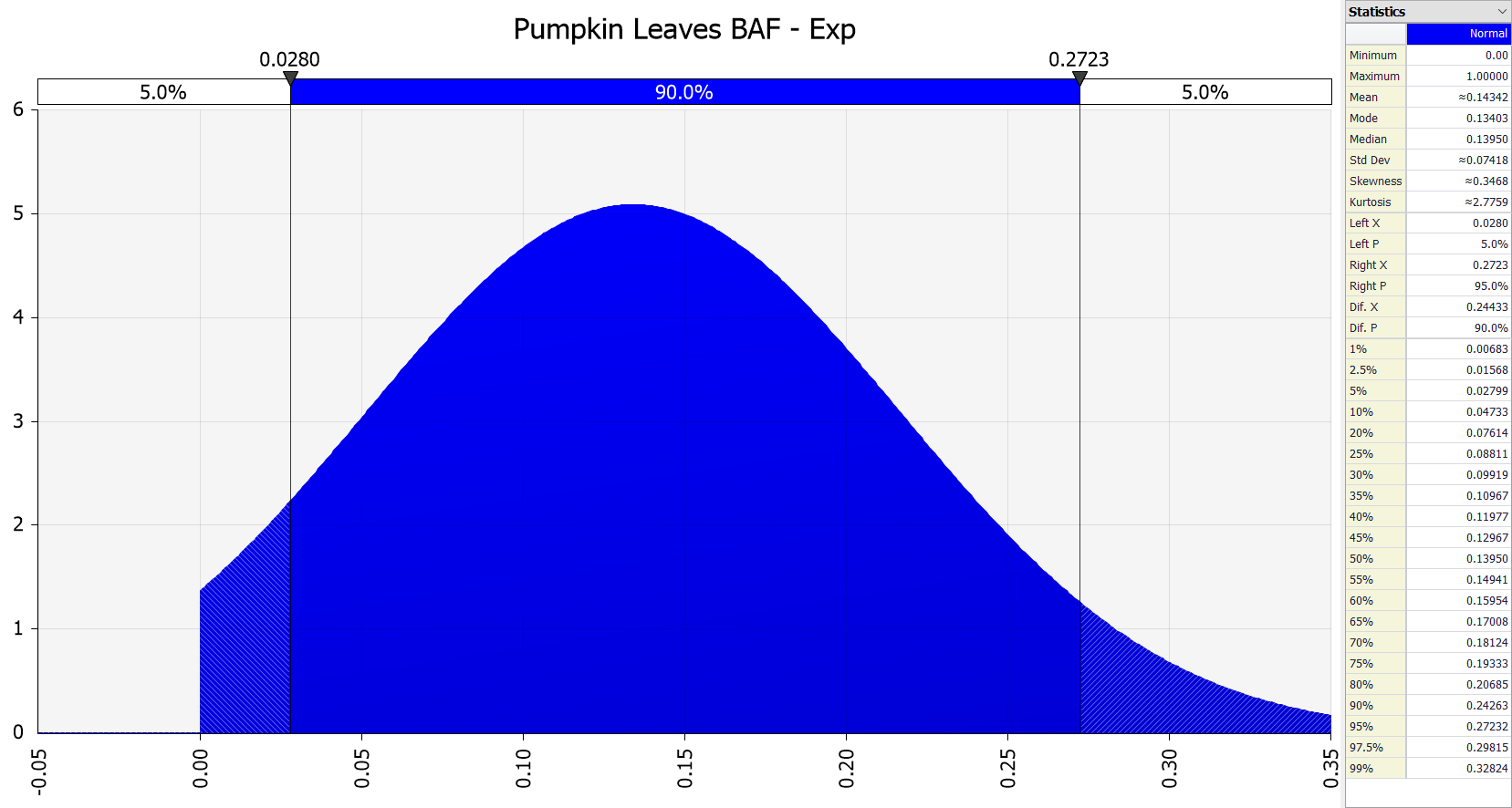


Figure S4. Probability distribution function for BAF_leaves_ based on site-specific data.

### Pumpkin BMP


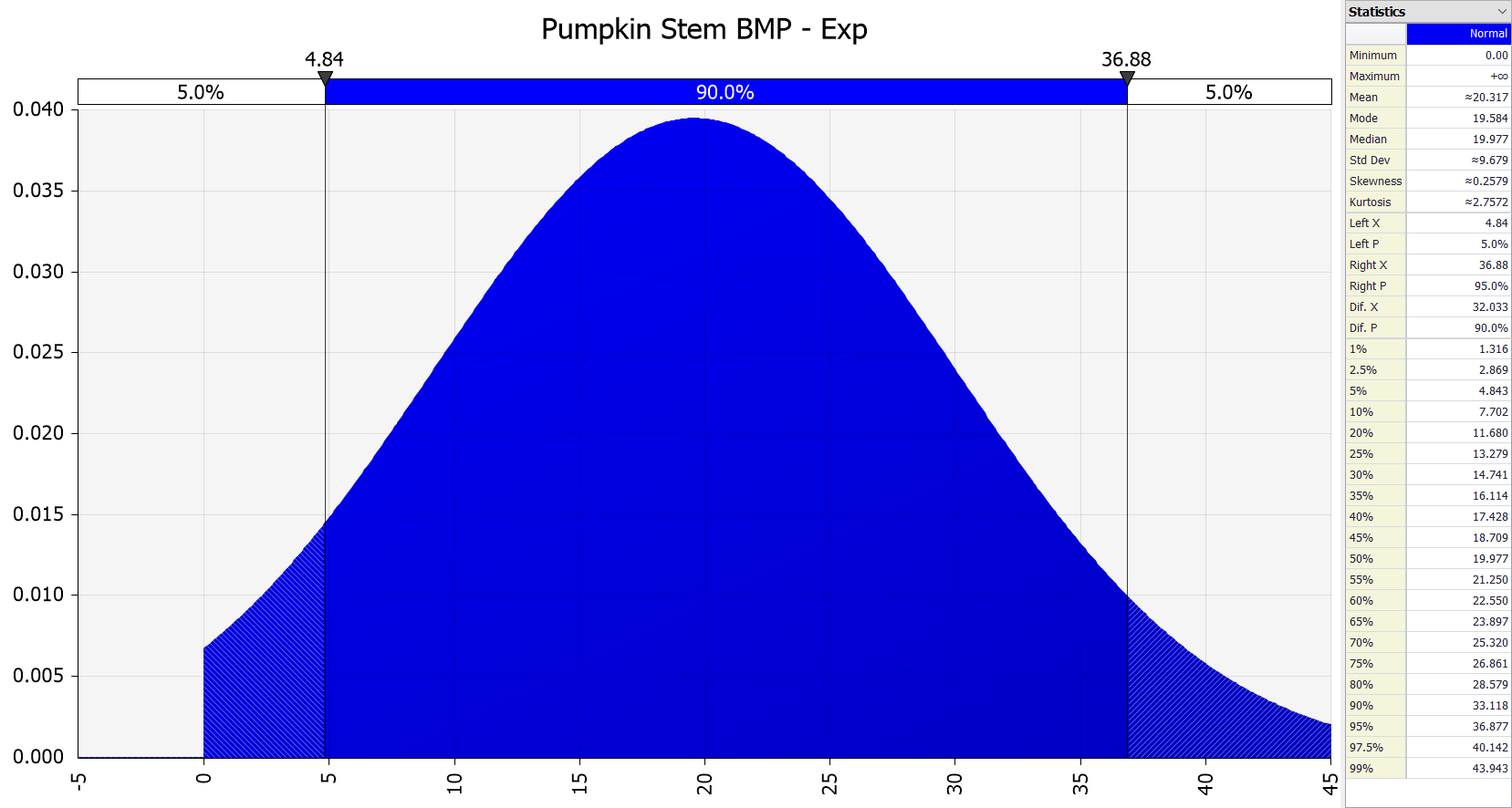


Figure S5. Probability distribution function for BMP_stem_ based on site-specific data. Note: the data is here presented for the mean stem dry weight per plant.


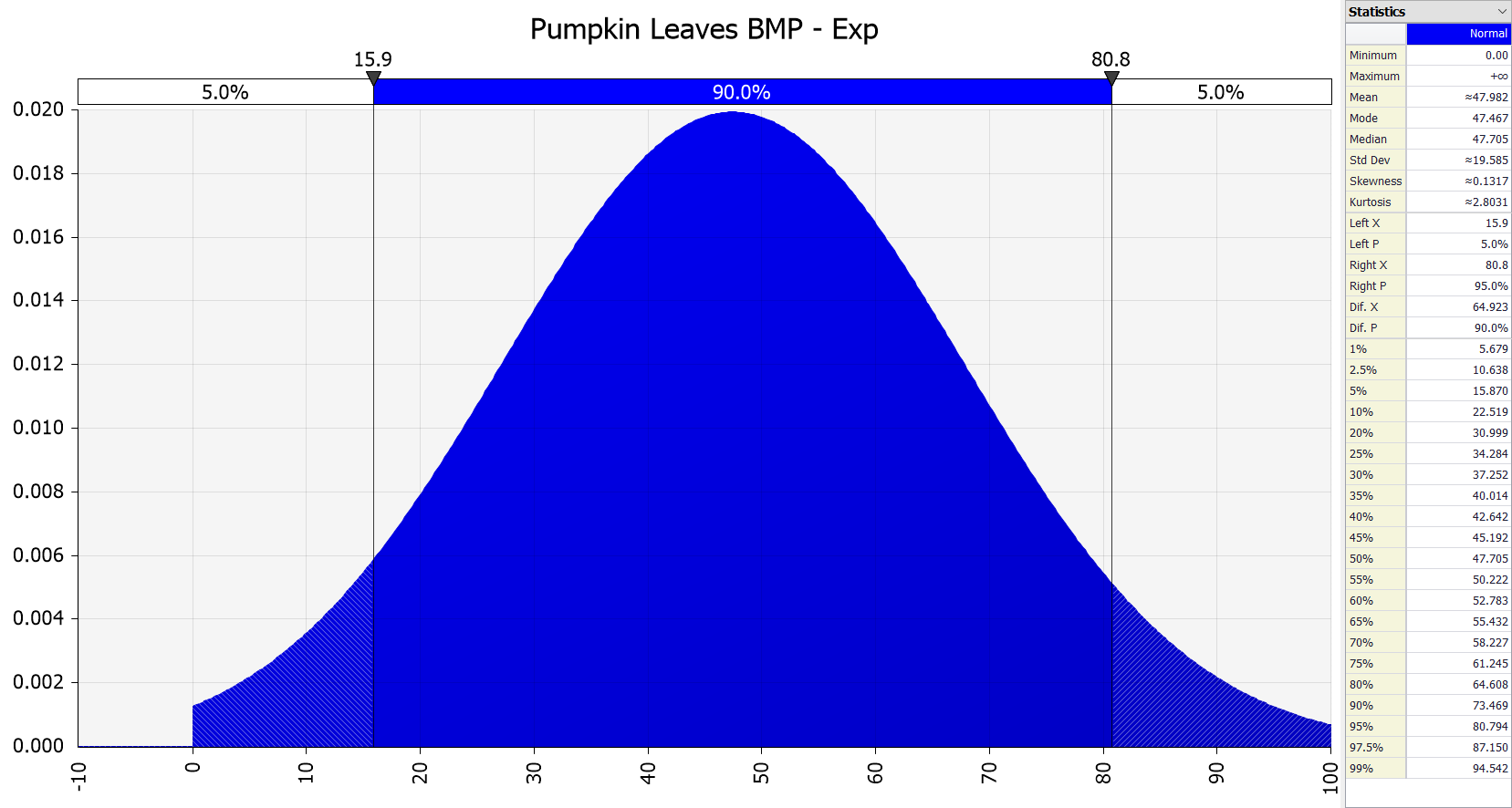


Figure S6. Probability distribution function for BMP_leaves_ based on site-specific data. Note: the data is here presented for the mean leaves dry weight per plant.

## Literature data

Empirical data from phytoextraction field experiments using pumpkin (*Cucurbita pepo* ssp. *pepo*, cv. Howden) was gathered during literature review to create a representative dataset for both *BAF* and *BMP*. The data was compiled and generalized by calculating average *BAFs* for ΣDDX and *BMP* per plant part and square area to create probability distributions for the variables.

### Pumpkin BAF


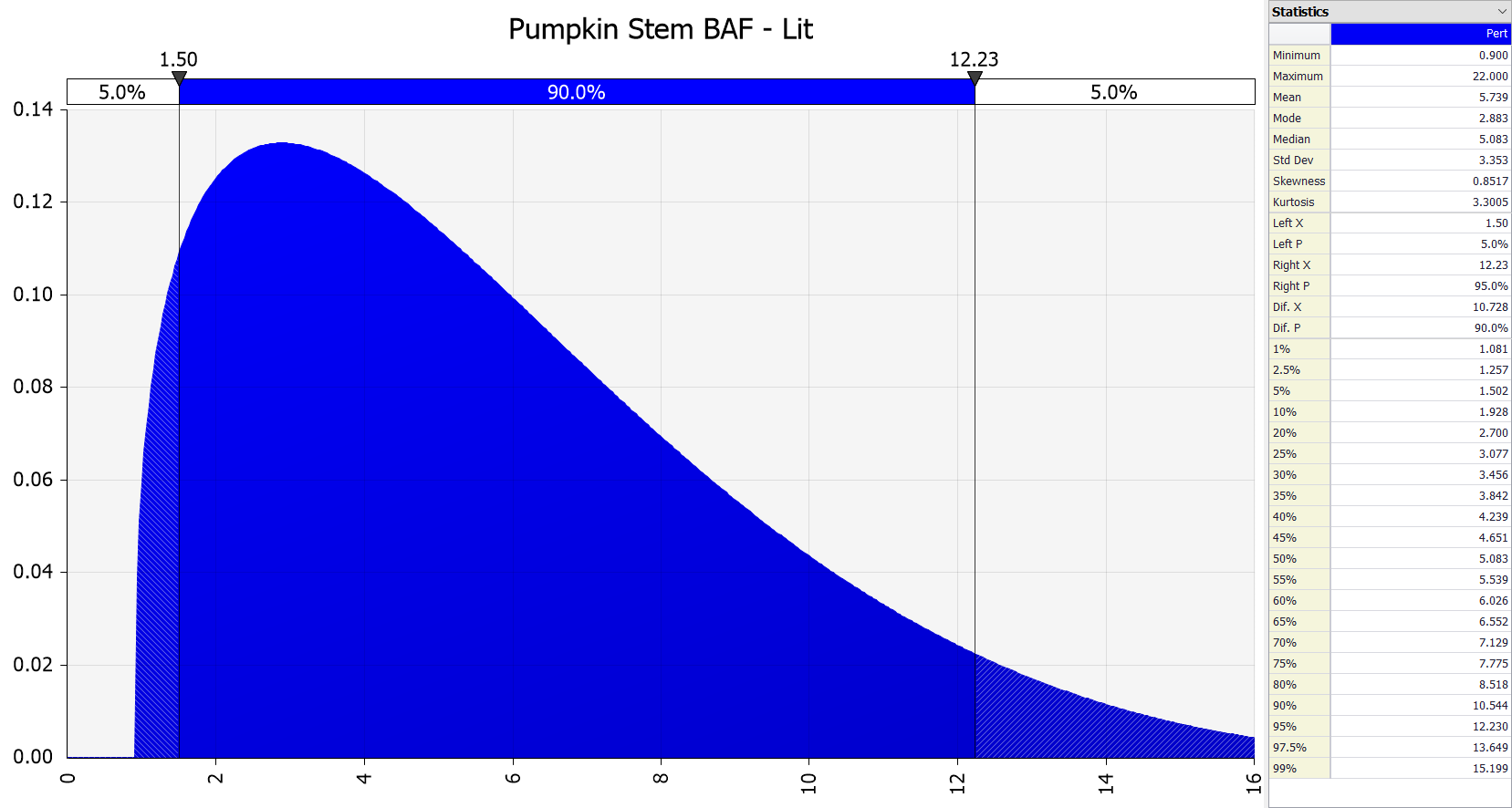


Figure S7. Probability distribution function for BAF_stem_ based on literature data.


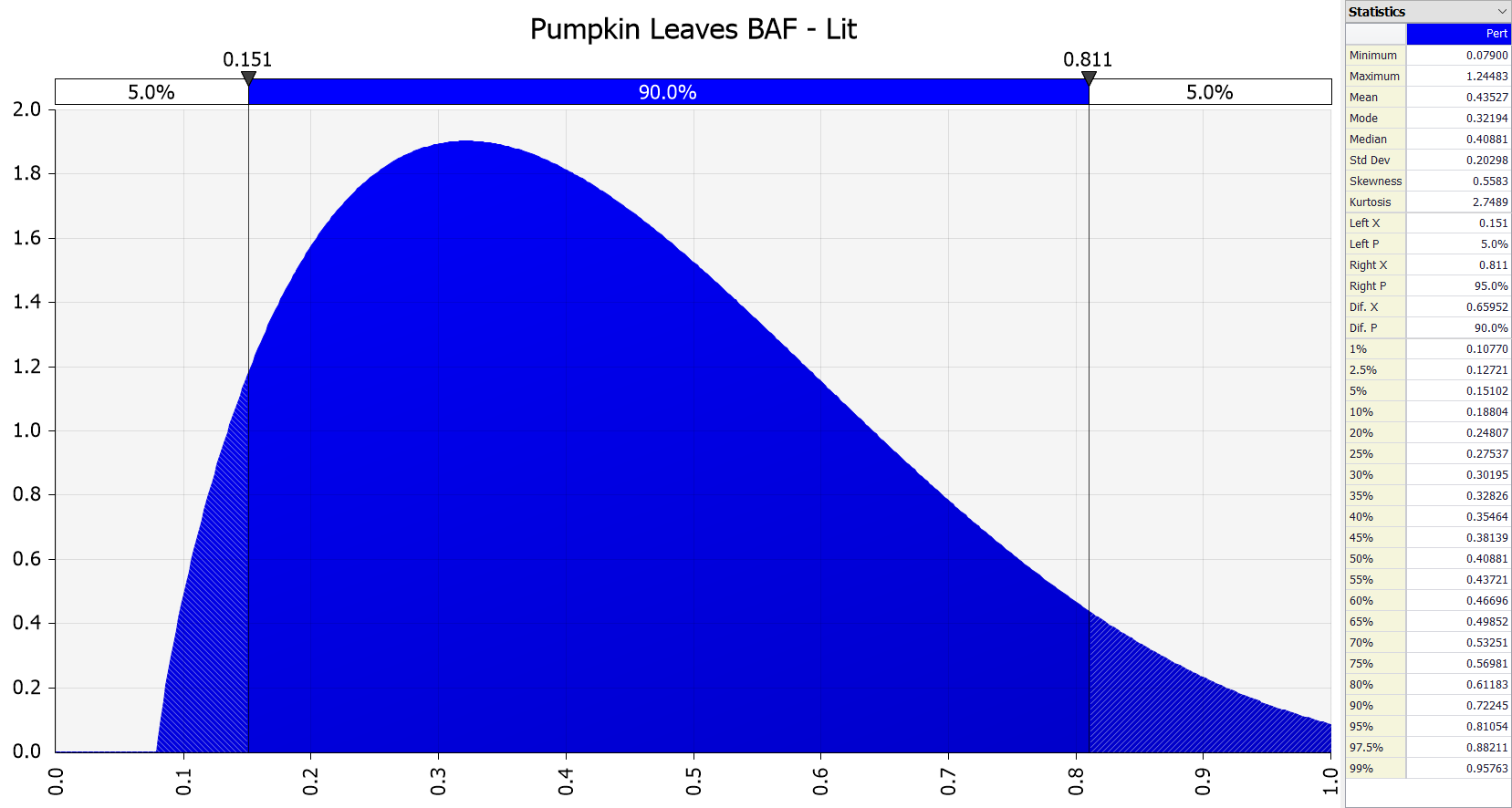


Figure S8. Probability distribution function for BAF_leaves_ based on literature data.

### Pumpkin BMP


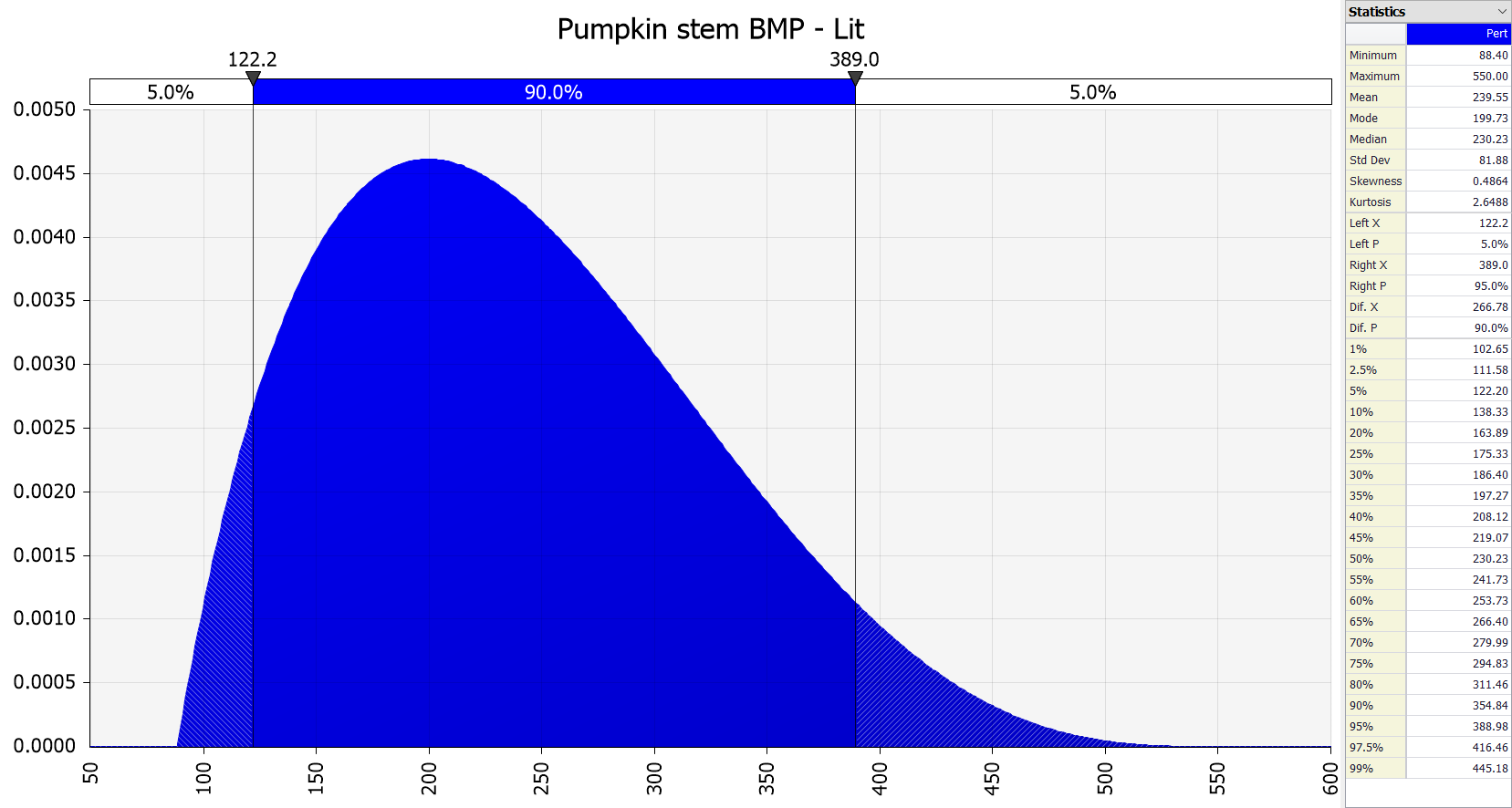
Figure S9. Probability distribution function for BMP_stem_ based on literature data. Note: the data is here presented for the mean stem dry weight per plant.


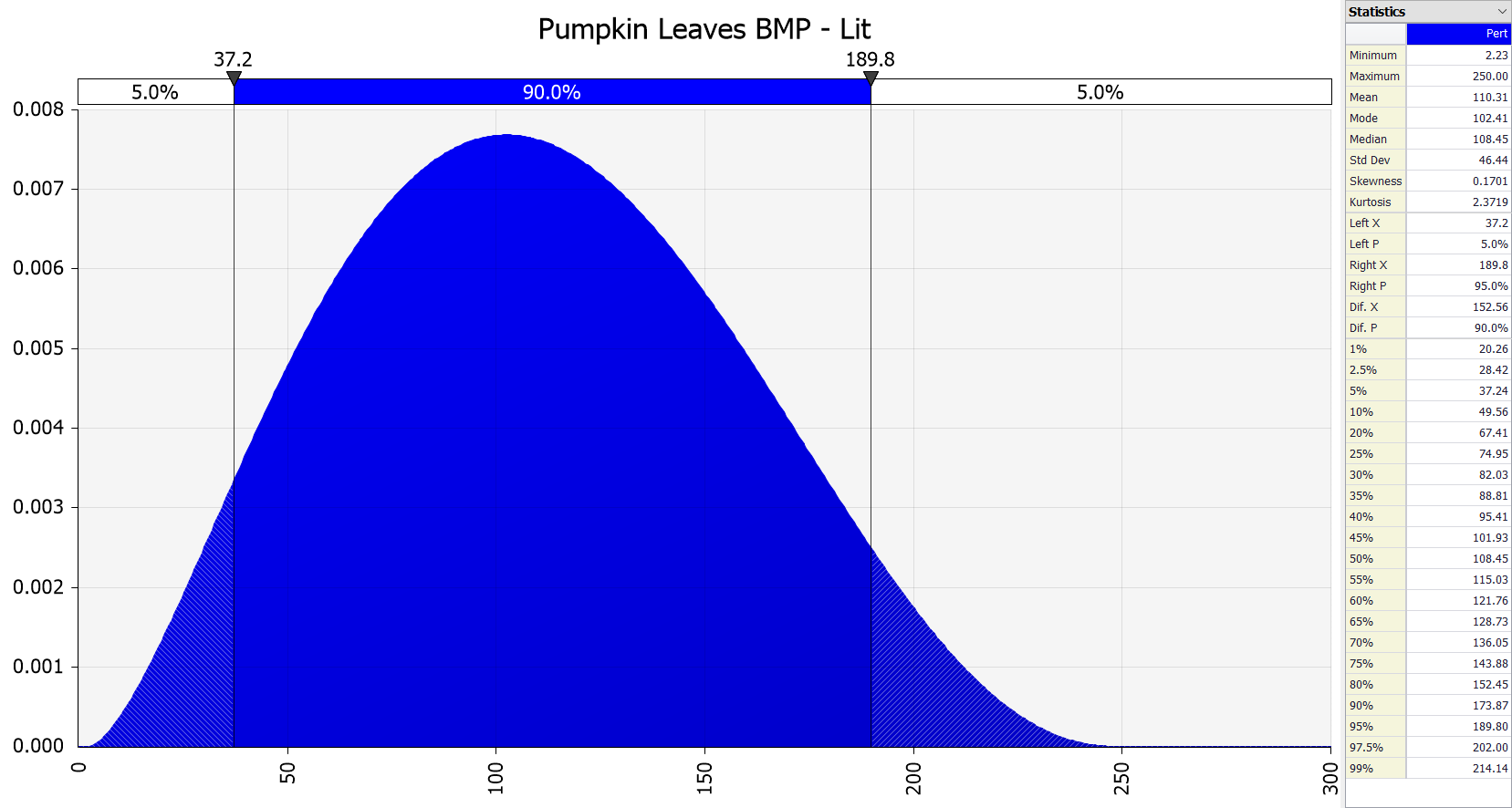
Figure S10. Probability distribution function for BMP_leaves_ based on literature data. Note: the data is here presented for the mean leaves dry weight per plant.

# Results and sensitivity analysis

Figures S11, S13, S15, and S17 show the resulting probability distribution functions for the simulations to estimate the time requirements for both literature and site-specific data using either the linear analytical model or the first-order exponential decay model.

Figures Figure S12, S14, S16, and S18 show the contribution of input variables (y-axis) to the total variance of the simulation result, time requirement, based on Spearman rank correlation. For this specific case, the negative coefficients for input variables indicate that the lower the value of the variable, the shorter the time required. The positive coefficients indicate that the higher the value of the variable, the longer the time required.

## Site-specific data


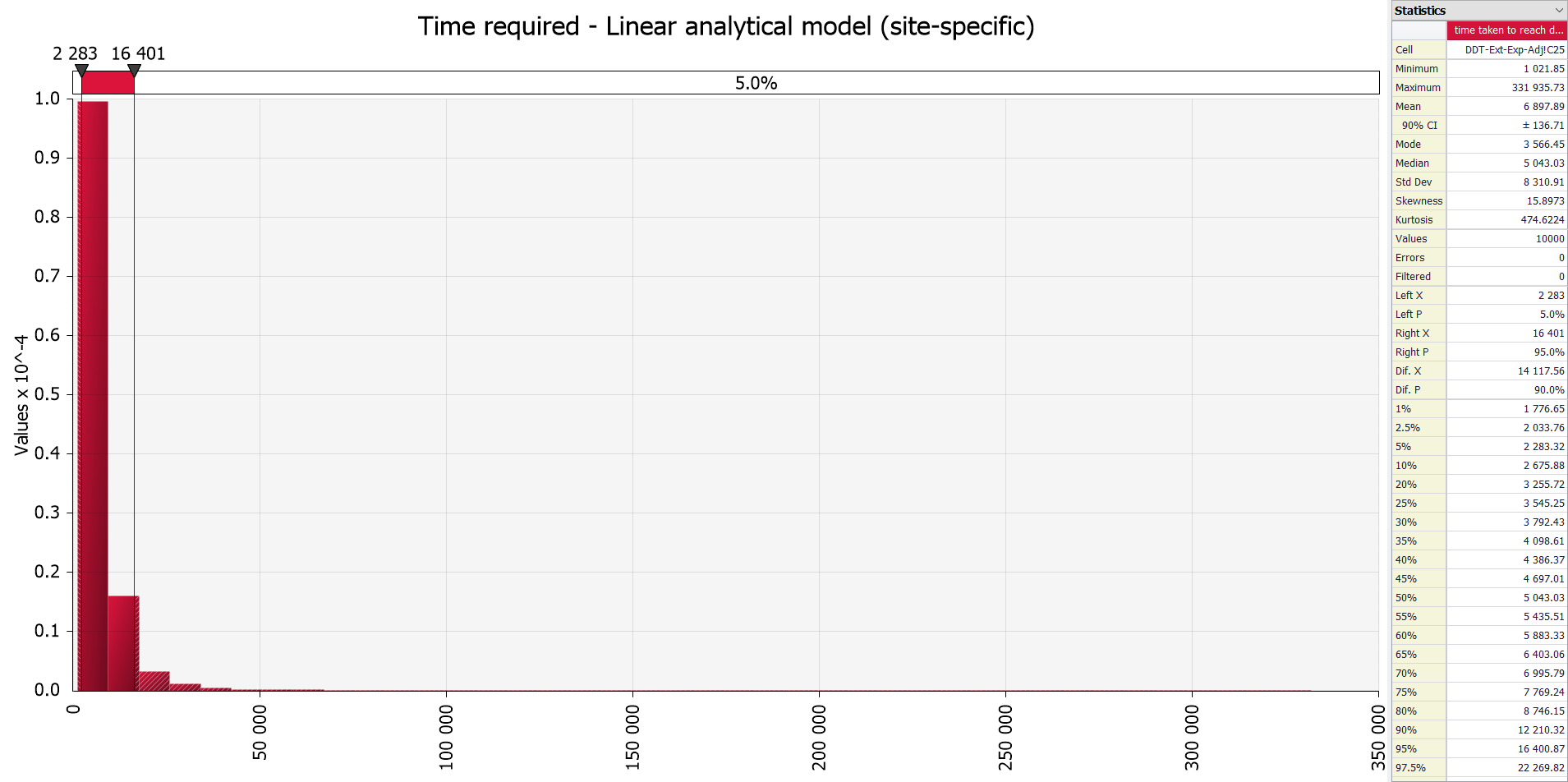


Figure S11. Probability distribution function for results of time requirement simulations for the linear analytical model based on site-specific data. The numbers at the top x-axis show the 5^th^ (left) and 95^th^ (right) percentile of the distribution.

Figure S12. Spearman rank correlation coefficients of input variables showing linear relationships between these input variables (y-axis) and the mean simulated time required. Results shown for the linear analytical model and site-specific data.


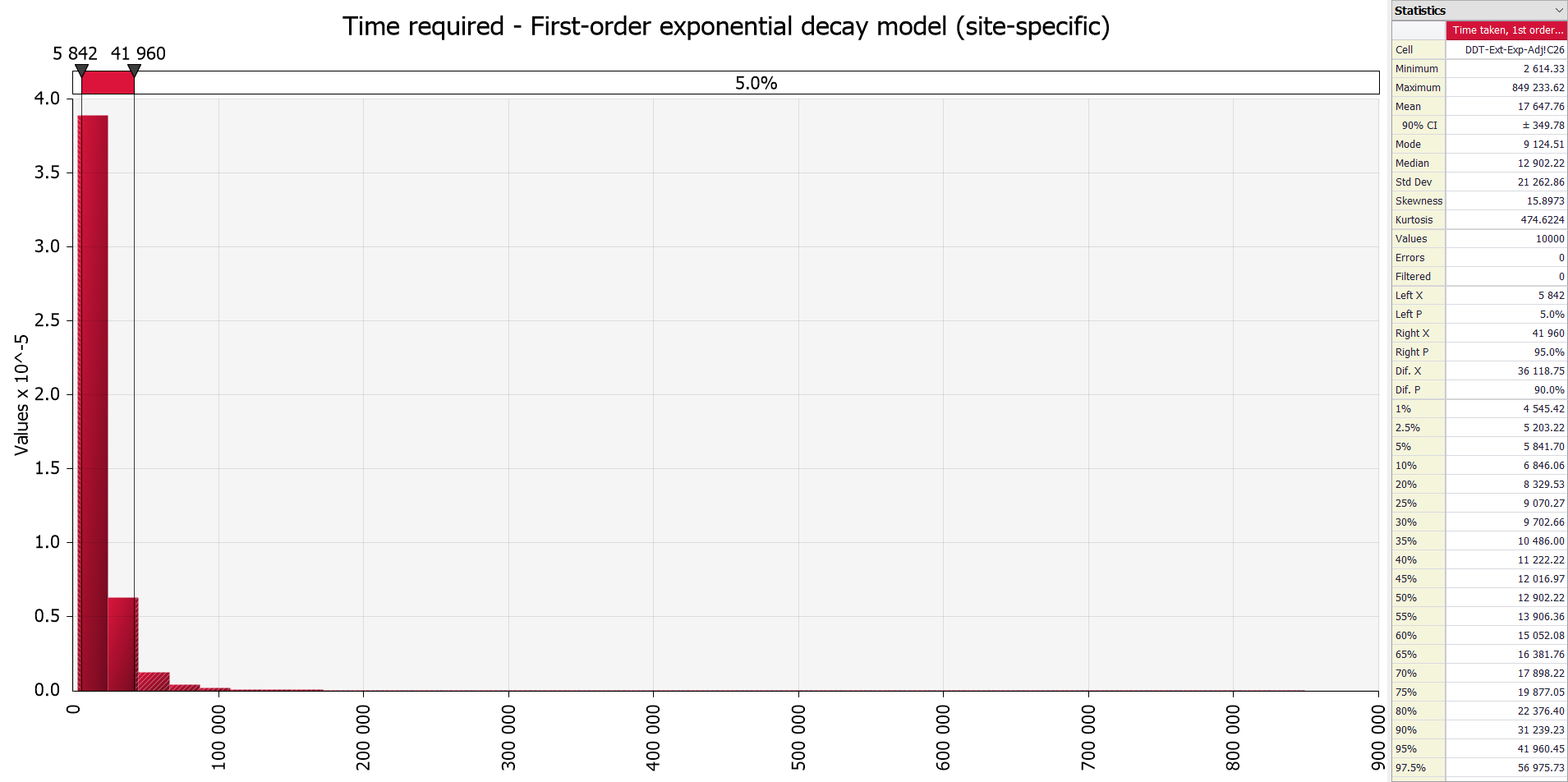


Figure S13. Probability distribution function for results of time requirement simulations for the first-order exponential decay model based on site-specific data. The numbers at the top x-axis show the 5^th^ (left) and 95^th^ (right) percentile of the distribution.

Figure S14. Spearman rank correlation coefficients of input variables showing linear relationships between these input variables (y-axis) and the mean simulated time required. Results shown for the first-order exponential decay model and site-specific data.

## Literature data


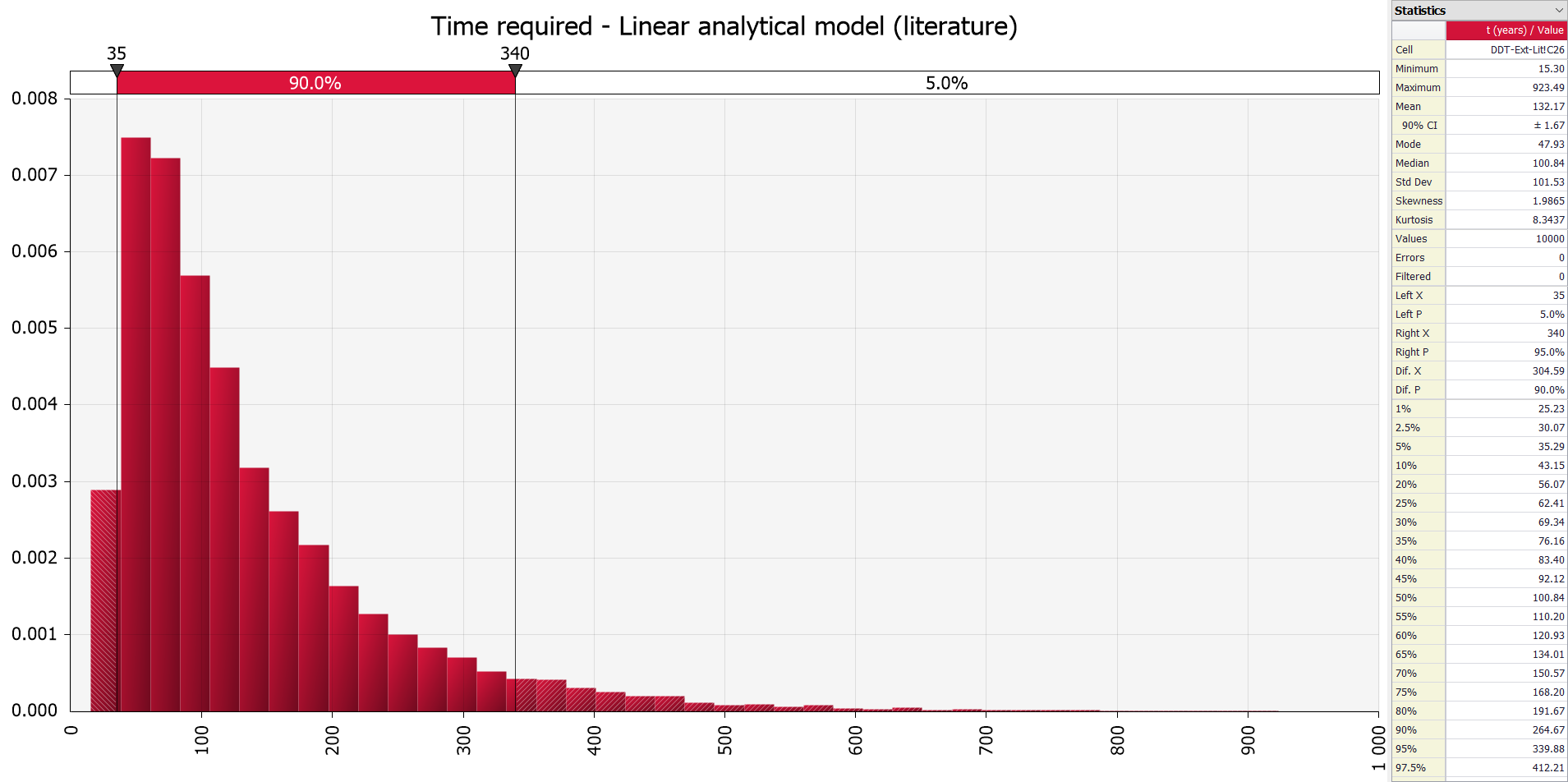


Figure S15. Probability distribution function for results of time requirement simulations for the linear analytical model based on literature data. The numbers at the top x-axis show the 5^th^ (left) and 95^th^ (right) percentile of the distribution.

Figure S16. Spearman rank correlation coefficients of input variables showing linear relationships between these input variables (y-axis) and the mean simulated time required. Results shown for the linear analytical model and literature data.


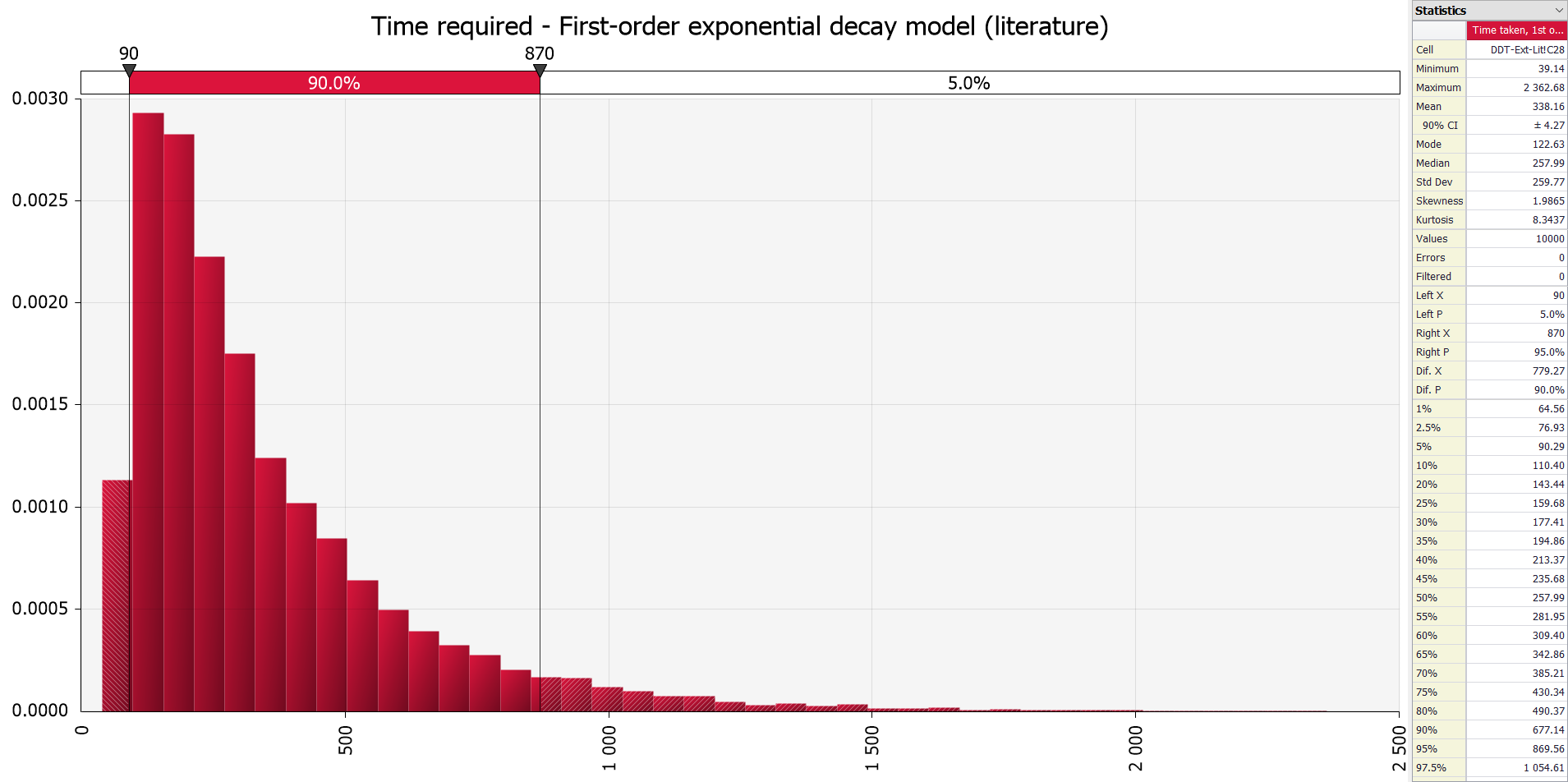


Figure S17. Probability distribution function for results of time requirement simulations for the first-order exponential decay model based on literature data. The numbers at the top x-axis show the 5^th^ (left) and 95^th^ (right) percentile of the distribution.

Figure S18. Spearman rank correlation coefficients of input variables showing linear relationships between these input variables (y-axis) and the mean simulated time required. Results shown for the first-order exponential decay model and literature data.
